# Differential exhumation of cratonic and non-cratonic lithosphere revealed by apatite fission-track thermochronology along the edge of the São Francisco craton, eastern Brazil

**DOI:** 10.1038/s41598-022-06419-w

**Published:** 2022-02-17

**Authors:** Ana Fonseca, Simone Cruz, Tiago Novo, Zhiyuan He, Johan De Grave

**Affiliations:** 1 Department of Geology, Ghent University, Ghent, Belgium; 2 Universidade Federal da Bahia, Instituto de Geociências, Salvador, BA Brazil; 3 Programa de Pós-Graduação Em Geologia, Universidade Federal de Minas Gerais, IGC-CPMTC, Belo Horizonte, Brazil

**Keywords:** Geodynamics, Geology, Tectonics

## Abstract

Lithosphere of cratons and orogens generally reacts differently to tectonic events. Although these differences are mostly clear during the orogenic phases, understanding how they respond to tectonic reactivation is still challenging. Here, we report the first detailed apatite fission-track (AFT) study pinpointing the gradual transition between cratonic and orogenic lithosphere, using the case study of the São Francisco craton (SFC) and the adjacent Araçuaí-West Congo Orogen (AWCO), eastern Brazil. The collision that built the AWCO partially affected the inherited rift structures of the Paramirim Aulacogen, embedded in the São Francisco-Congo paleocontinent. Our data reveal a differential Phanerozoic exhumation between closely interspaced areas affected and not affected by the AWCO deformation. Samples from the SFC present slow and protracted basement cooling during the Phanerozoic, while samples from the orogen display rapid exhumation since the Eocene. An intermediate ~ N–S zone of c.40 km shows lower magnitude basement cooling during the Cenozoic, possibly because the propagation of AWCO deformation decreases towards the craton interior. Within the orogen, the Rio Pardo salient is the main reactive structure and probably results from the deformation of a master fault, inherited from its precursor rift. Here, we show how the magnitude of Phanerozoic denudation may be deeply associated with previous events of lithosphere weakening.

## Introduction

**Figure 1:**
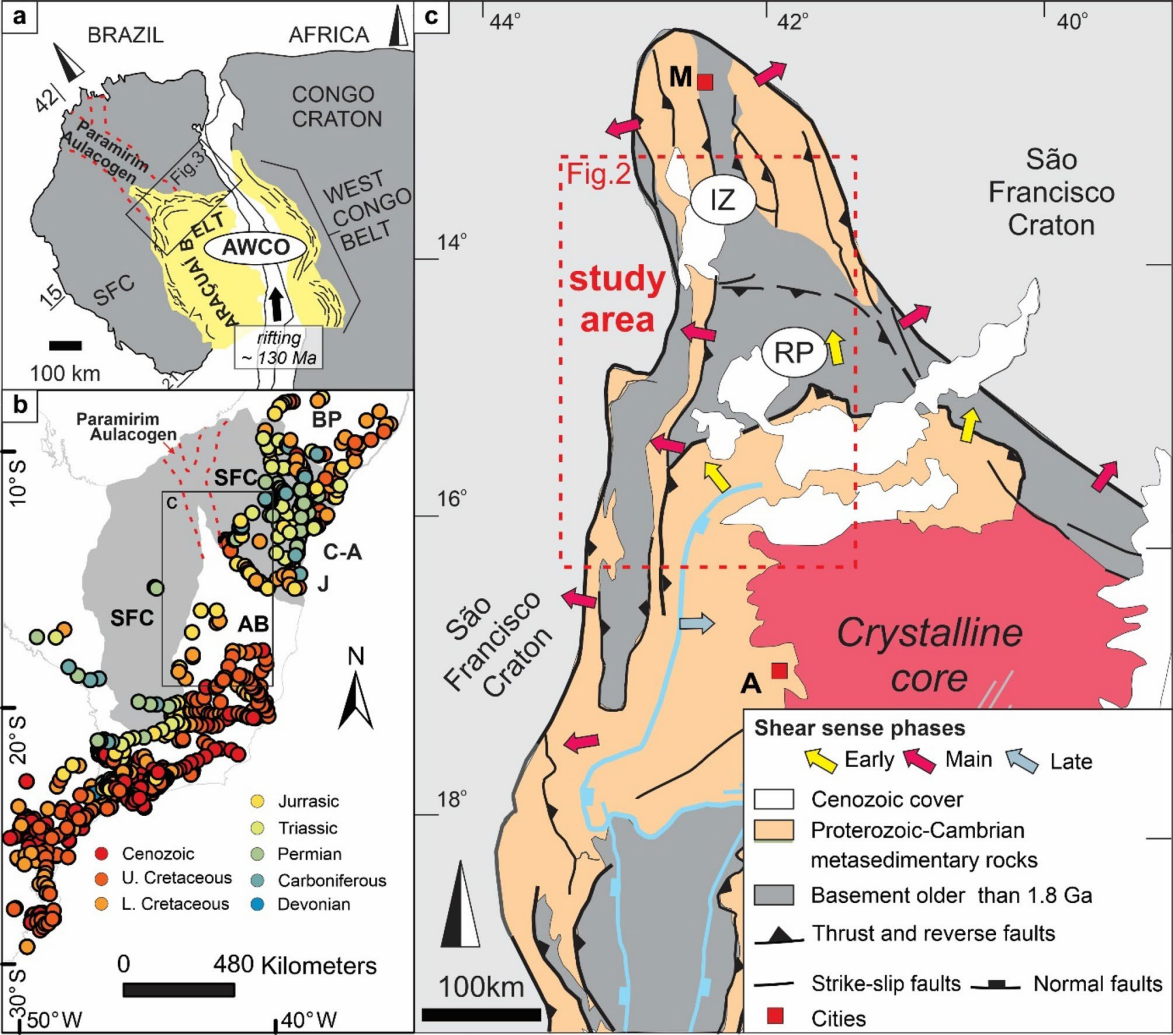
Geological context of the study area. (**a**) The Araçuaí-West Congo Orogen (AWCO) and adjacent São Francisco-Congo craton in the context of West Gondwana. (**b**) Published AFT ages^4^. *SFC* São Francisco craton, *AB* Araçuaí belt, *BP* Borborema province, *C-A* Camamu/Almada basin, *J* Jequiqinhonha basin. (**c**) Simplified geologic map of the northern AB and the high grade/granitic core of the AWCO^7^. *IZ* Interference zone, *RP* Rio Pardo salient. *Cities A* Araçuaí, *M* Macaúbas. The maps were created using Corel Draw Graphics Suite 2018 (http://www.coreldraw.com).

Cratons and orogens reflect two distinct types of lithospheres, characterized by their different genesis and by their response to posterior tectonic history. Orogenic lithosphere is generally easily reactivated and reworked, while the cold and rigid cratonic lithosphere is more resilient to major tectonic events^1,2^. In Brazil, cratons and intervening orogens compose the current passive Atlantic margin, where they are shown to have different responses to the Cretaceous rifting of the South Atlantic Ocean and later tectonic stresses^3,4^. Initial rifting during West Gondwana break-up eventually separated the South American plate (Brazil) from its (West) African counterpart. The two conjugate Atlantic passive margins further remained in distal positions with respect to tectonically active regions^5^. Considering the large dataset of apatite-fission track (AFT) thermochronology from the Brazilian passive margin, it was possible to verify that, for example, samples from the São Francisco craton (SFC) mainly yield AFT ages older than the Early Cretaceous, i.e. pre-rift (Fig. 1), whereas, in the adjacent orogens, i.e. Araçuaí belt (AB) and Borborema Province, AFT ages tend to be younger than the Early Cretaceous, i.e. rift to post-rift (Fig. 1). These results point to a first-order influence of lithosphere composition and properties with respect to basement exhumation^4^.

Orogenic phases may rework cratonic lithosphere at its borders, mainly by thrusting and folding of the cratonic marginal terranes or by partial inversion of aulacogen structures in the craton. While the SFC remained weakly deformed, the northern half of the AB, in eastern Brazil, was generated by the inversion of pre-existent NNW-trending rift structures and nucleation of new shear zones by WSW–ENE compression during the Ediacaran–Cambrian orogeny^6,7^. The objective of our study is to gain deeper insights into the exhumation of the basement in an area of transition between the SFC and the AB, during subsequent Phanerozoic tectonic events and, thus, verify how different types of lithosphere exert control on later basement exhumation.

## Geological background

**Figure 2:**
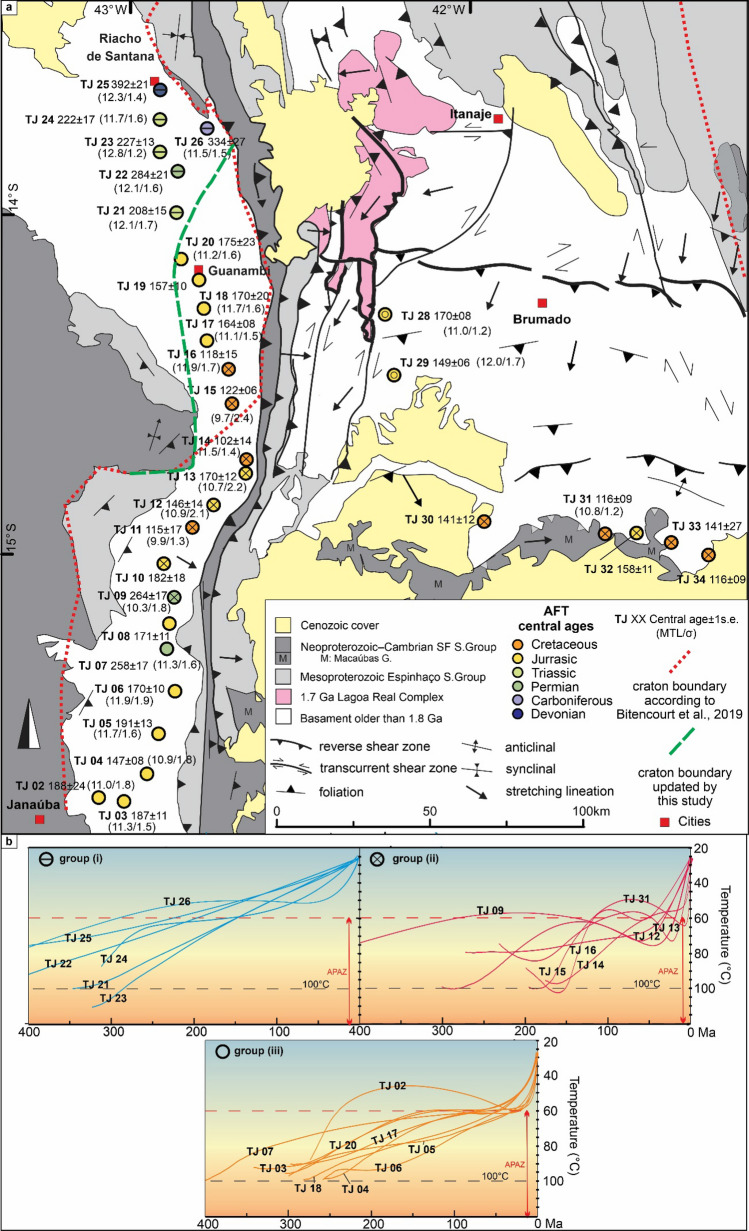
(**a**) Geological map of the study area (modified from^8^) with indication of sample locations, central AFT ages (in Ma), Mean Track Lengths (MTL) (in μm), and MTL standard deviation. (**b**) Expected t-T paths from thermal history modelling (QTQt^10^; individual data in Supplementary S.1 online), grouped by thermal history trend. The map and charts were created using Corel Draw Graphics Suite 2018 (http://www.coreldraw.com) and ArcGIS 10.4.1 (https://www.esri.com).

The São Francisco craton (SFC) consists of an Archean-Paleoproterozoic basement older than 1.8 Ga, with Mesoproterozoic to Cambrian cover units^8^(Fig. 2). To the southeast of the craton, the Araçuaí belt (AB) involving a basement older than 1.8 Ga and Proterozoic-Cambrian cover complexes, encompasses two large scale features: (i) a large arcuate salient, the Rio Pardo Salient, and (ii) a NNW-trending so-called interference zone (IZ in the Fig. 1) with a subparallel oriented Proterozoic intracontinental rift structure, the Paramirim Aulacogen^8^ (Figs. 1 and 2). The interference zone is characterized by the inversion of the normal faults of the aulacogen phase and nucleation of thrusts, folds, and dextral shear zones. The AB, as a whole, is a segment of the wider Araçuaí-West Congo Orogen (AWCO) (Fig. 1), which developed along the margin of the SFC and the Congo Craton during the Ediacaran to Early Cambrian (^9^ and references therein). The AWCO evolved due to the closure of an embayment, i.e. the terminal branch of the Adamastor ocean, carved into the São Francisco-Congo paleocontinent, and eventually resulted in the assembly of West Gondwana^9^.

**Figure 3:**
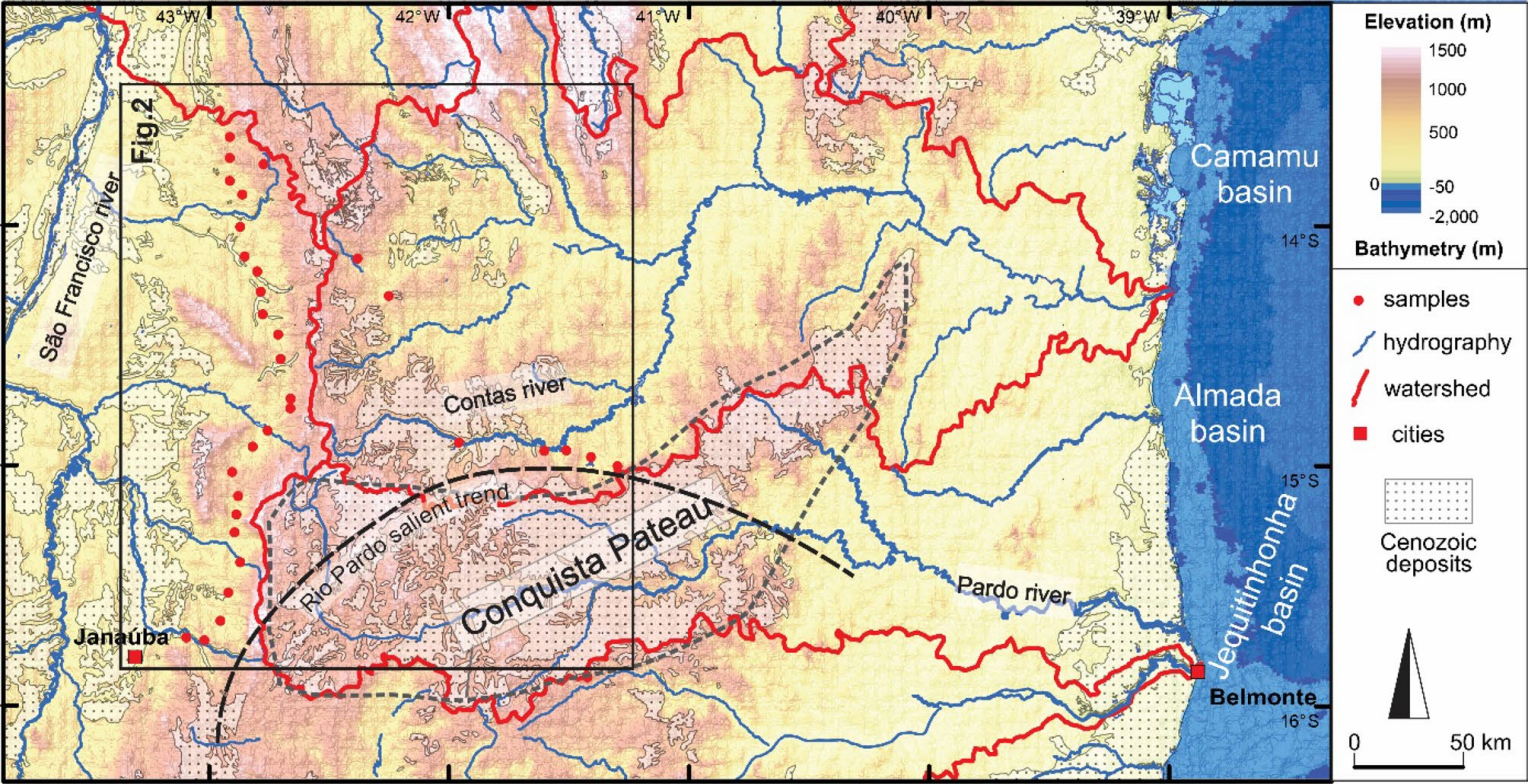
Topographic and bathymetric map of the study area and adjacent passive margin. Source of the digital elevation model was USGS (https://earthexplorer.usgs.gov). Source of hydrography data was ANA (https://dadosabertos.ana.gov.br/). The map was created using, ArcGIS 10.4.1 (https://www.esri.com) and Corel Draw Graphics Suite 2018 (http://www.coreldraw.com).

The SFC and the AB remained confined in the interior of West Gondwana from the early Paleozoic until the rifting and break-up of this supercontinent around 130 Ma^5^. The separation between South America and Africa took place with the opening of the South Atlantic Ocean and resulted in the shifting of the regional base level of our study area towards the present-day coast. Currently, the Contas River drains the area to the east, while the São Francisco River drains it to the north (Fig. 3). Erosion-resistant quartzite ridges from the Espinhaço Supergroup (Figs. 2 and 3) form the N-S oriented watershed that reaches up to 1400 m altitude. Diffuse and thin (meters thick) Cenozoic deposits are present in the main river channels and laterized plateaus, mainly in the Conquista Plateau (Fig. 3). This plateau is also relatively elevated to around 1000 m and partially overlaps the Rio Pardo salient structures, where it delimits the the Contas River Basin to the south.

### Apatite fission-track thermochronology: method and results

Thirty-two basement samples (gneiss, granites and migmatites) were collected from the basement of the AB–SFC boundary (Fig. 2) to perform low-temperature thermochronology with the AFT method. In order to explore the effects of northwards decreasing deformation, we collected closely interspaced samples in an N–S transect, crossing the limit orogen/craton. Another transect was sampled in the Rio Pardo salient structure. Sample lithology, locations, and elevations are found in Table 1.

**Table 1:** Summary of AFT analyses with sample location (in World Geodetic System -WGS84), elevation (Z) and lithology.

Sample	X (°)	Y (°)	Z (m)	Lithology	n	ρ_s_ (± 1σ)	N_s_	ρ_i_ (± 1σ)	N_i_	ρ_d_ (± 1σ)	N_d_	P(χ^2^)	AFT central age (Ma)	1 s.e. (AFT age)	n_c_	MTL (µm)	σ (MTL) (µm)	Skew-ness	D_par_ (µm)
TJ 02	− 15.7176	− 43.0938	583	gneiss	20	10.961(0.314)	1213	3.740(0.181)	427	4.059(0.090)	2048	0.76	188	24	99	11.3	1.5	− 0.165	1.45
TJ 03	− 15.7293	− 43.0192	598	mylonitic granite	20	14.283(0.388)	1358	5.110(0.233)	479	4.055(0.090)	2045	0.87	187	11	100	11.0	1.8	− 0.432	1.78
TJ 04	− 15.6480	− 42.9518	524	porphyritic granite	20	12.044(0.339)	1260	5.420(0.228)	566	4.050(0.090)	2043	0.97	147	8	100	10.9	1.8	− 0.389	1.85
TJ 05	− 15.5306	− 42.9195	589	gneiss	20	11.267(0.344)	1072	3.963(0.206)	369	4.044(0.090)	2040	0.97	191	13	100	11.7	1.6	− 0.236	2.61
TJ 06	− 15.4044	− 42.8690	563	gneiss	20	13.567(0.377)	1294	5.212(0.233)	502	4.040(0.090)	2037	0.97	170	10	100	11.9	1.9	− 1.048	2.93
TJ 07	− 15.2799	− 42.8929	536	gneiss	20	11.717(0.321)	1335	3.207(0.165)	338	4.035(0.090)	2034	0.94	258	17	65	11.3	1.6	− 0.213	2.30
TJ 08	− 15.2058	− 42.8859	578	gneiss	20	11.952(0.386)	960	4.549(0.236)	370	4.030(0.090)	2032	0.88	171	11	−	−	−	−	1.73
TJ 09	− 15.1282	− 42.8782	645	gneiss	20	13.463(0.343)	1540	3.402(0.175)	378	4.006(0.089)	2018	0.47	264	17	50	10.3	1.8	0.547	2.29
TJ 10	− 15.0288	− 42.9024	612	gneiss	20	3.529(0.168)	442	1.288(0.102)	159	4.001(0.089)	2016	0.67	182	18	−	−	−	−	1.94
TJ 11	− 14.9223	− 42.8181	570	gneiss	20	7.152(0.223)	1027	4.066(0.168)	587	4.000(0.089)	2013	0.32	115	7	24	9.9	1.3	− 0.649	1.65
TJ 12	− 14.8557	− 42.7556	570	pegmatitic granite	20	18.428(0.395)	2180	8.167(0.262)	971	3.992(0.089)	2010	0.56	146	14	100	10.9	2.1	− 0.114	1.88
TJ 13	− 14.7631	− 42.6609	684	filonite	20	8.082(0.260)	967	3.076(0.160)	368	3.987(0.089)	2008	0.42	170	12	80	10.7	2.2	− 0.044	2.25
TJ 14	− 14.7226	− 42.6589	651	granite	20	6.919(0.220)	987	4.469(0.176)	643	3.982(0.089)	2005	0.12	102	14	65	11.5	1.4	− 0.551	1.83
TJ 15	− 14.5580	− 42.7015	612	granite	20	17.514(0.430)	1662	9.395(0.316)	886	3.977(0.089)	2002	0.85	122	6	98	9.7	2.4	0.214	1.82
TJ 16	− 14.4564	− 42.7112	583	mafic gneiss	20	31.780(0.725)	1920	17.523(0.539)	1056	3.972(0.089)	2000	0.83	118	5	100	11.9	1.7	0.020	1.80
TJ 17	− 14.3723	− 42.7756	562	migmatite	20	33.574(0.688)	936	13.544(0.442)	2379	3.953(0.089)	1989	0.67	164	8	100	11.1	1.5	− 0.197	1.81
TJ 18	− 14.2777	− 42.7839	520	porphyritic granite	20	24.118(0.587)	1687	9.297(0.368)	638	3.949(0.089)	1986	0.32	170	20	100	11.7	1.6	− 0.070	2.25
TJ 19	− 14.1933	− 42.7989	515	granite	20	8.228(0.257)	1028	3.334(0.162)	421	3.944(0.089)	1984	0.77	157	10	−	−	−	−	1.59
TJ 20	− 14.1316	− 42.8511	521	granite	20	25.679(0.676)	1445	9.475(0.410)	533	3.939(0.089)	1981	0.16	175	23	100	11.2	1.6	0.241	1.72
TJ 21	− 14.0331	− 42.7132	546	granite	20	11.472(0.370)	962	3.540(0.206)	296	3.934(0.088)	1978	0.86	208	15	100	12.1	1.7	0.472	1.68
TJ 22	− 13.8732	− 42.8608	538	migmatitic gneiss	20	11.461(0.335)	1170	2.543(0.157)	262	3.930(0.088)	1976	0.95	284	21	56	12.1	1.6	0.016	1.85
TJ 23	− 13.8162	− 42.9137	543	migmatitic gneiss	20	27.629(0.687)	1619	7.644(0.358)	455	3.925(0.088)	1973	0.88	227	13	100	12.8	1.2	0.241	2.29
TJ 24	− 13.7204	− 42.9133	572	Gneiss	20	6.864(0.239)	828	1.897(0.123)	238	3.920(0.088)	1970	0.76	222	17	53	11.7	1.6	0.313	1.79
TJ 25	− 13.6337	− 42.9129	634	granite	20	25.076(0.481)	2714	5.181(0.219)	559	5.044(0.101)	2503	0.65	392	21	100	12.3	1.4	0.020	2.46
TJ 26	− 13.7463	− 42.7732	816	migmatitic gneiss	21	8.387(0.283)	878	2.092(0.143)	213	5.031(0.101)	2496	0.83	334	27	100	11.5	1.5	− 0.073	1.94
TJ 28	− 14.2947	− 42.2507	620	mylonite	26	34.889(0.741)	2218	16.799(0.514)	1068	5.004(0.100)	2483	0.16	170	8	28	11.0	1.2	− 0.215	1.57
TJ 29	− 14.4735	− 42.2246	586	granite	20	28.712(0.568)	2553	15.686(0.420)	1395	4.991(0.100)	2477	0.26	149	6	100	12.0	1.7	− 0.807	1.62
TJ 30	− 14.9048	− 41.9593	651	gneiss	27	2.657(0.127)	435	1.559(0.098)	251	4.978(0.100)	2471	1.00	141	12	–	–	–	–	1.60
TJ 31	− 14.9409	− 41.6027	540	mylonite	23	35.044(0.646)	2942	24.592(0.541)	2068	4.964(0.100)	2464	0.24	116	9	100	10.8	1.2	− 0.007	1.32
TJ 32	− 14.9389	− 41.5109	536	mylonite granite	20	6.625(0.239)	771	3.380(0.170)	395	4.951(0.099)	2457	0.64	158	11	–	–	–	–	1.52
TJ 33	− 14.9665	− 41.4084	515	gneiss	12	1.167(0.136)	74	0.628(0.135)	42	4.888(0.099)	2427	1.00	141	27	–	–	–	–	–
TJ 34	− 15.0034	− 41.2983	635	mylonite	20	3.981(0.178)	502	2.670(0.143)	346	4.876(0.099)	2421	0.90	116	9	–	–	–	–	1.69

N is the number of analyzed grains. ρ_s_ and ρ_i_ are the densities of spontaneous (apatite) and induced tracks (in muscovite external detector), respectively. ρ_d_ are interpolated values of the density of induced tracks in the external detector irradiated against regularly spaced Uranium-doped glass dosimeters. All densities expressed in 10^5^ tracks/cm^2^. N_s_ and N_i_ are the number of spontaneous and induced tracks, respectively. N_d_ is the interpolated value of the number of induced tracks in the external detector stemming from co-irradiated glass dosimeters. P(χ^2^) is the chi-squared probability that the dated grains have a constant ρ_s_/ρ_i_ ratio. The standard error (s.e.) on the age is given in Ma. N_c_ is the number of measured confined natural sub-horizontal tracks. MTL is the mean track length, σ is the standard deviation of the track length distribution.

The AFT method is a low-temperature dating technique based on the accumulation of mineral lattice damage, i.e. ‘fission tracks’, generated by the spontaneous fission of ^238^U^11^. These tracks are preserved in the apatite lattice on geological time scales at temperatures lower than c.120 °C, i.e. upper crustal temperatures^12^. AFT ages are hence cooling ages registering the time since the fission tracks became thermally stable in the apatite crystals. After etching to reveal the natural or spontaneous tracks by optical microscopy, the observed track density per unit area is a measure for the AFT age^11^. At temperatures between c.120–60 °C, fission tracks are able to accumulate in the apatite lattice but are subject to track length shortening due to thermal annealing or lattice restoration. Hence, a track length distribution of a sample is an indicator of the thermal history experienced by the apatite and its host rock^12,13^.

Apatite grains were concentrated using standard procedures, hand-picked and embedded in epoxy resin^14^. Mounts with c.120 apatite grains were etched for 20 s in 5.5 M HNO_3_ solution at 21 °C to reveal spontaneous fission tracks^15^. In this study we applied the external detector (ED) approach using thermal neutron irradiation^16^. U-free mica (Goodfellow, clear ruby) was attached as ED on top of each sample and age standard (Durango and Fish Canyon Tuff) mount^17^. IRMM-540 dosimeter glasses were used for monitoring the thermal neutron fluence. The packages were irradiated at the Belgian Nuclear Research Centre (SCK, Mol) using the Belgian Reactor 1 (BR1) facility (Channel X26;^18^). After irradiation, the ED was etched using 40% HF for 40 min at 21 °C in order to reveal induced fission tracks.

For each sample, 20 or more apatite grains were analysed. Fission track density was measured using a motorized Nikon Eclipse Ni-E microscope with a DS-Ri2 camera attached, at a 1000 × magnification. Central age calculation was performed using “IsoplotR”^19^ with an overall weighted mean zeta value of 330.6 ± 3.9 a·cm^2^ (Analyst AF) based on multiple Durango and Fish Canyon Tuff apatite age standards and the IRMM-540 dosimeter glass^20^. All samples pass the χ^2^ test, indicating single age populations. In 23 samples, where it was possible to achieve a representative AFT length–frequency histogram (n > 50), Markov Chain Monte Carlo (MCMC) inverse modeling was performed using the QTQt software^10^. Except for the present-day temperature (25 ± 15 °C), no time–temperature constraints were added. The Ketcham annealing model^21^, with D_par_ as the kinetic parameter was used. The data is summarized in Table 1.

**Figure 4:**
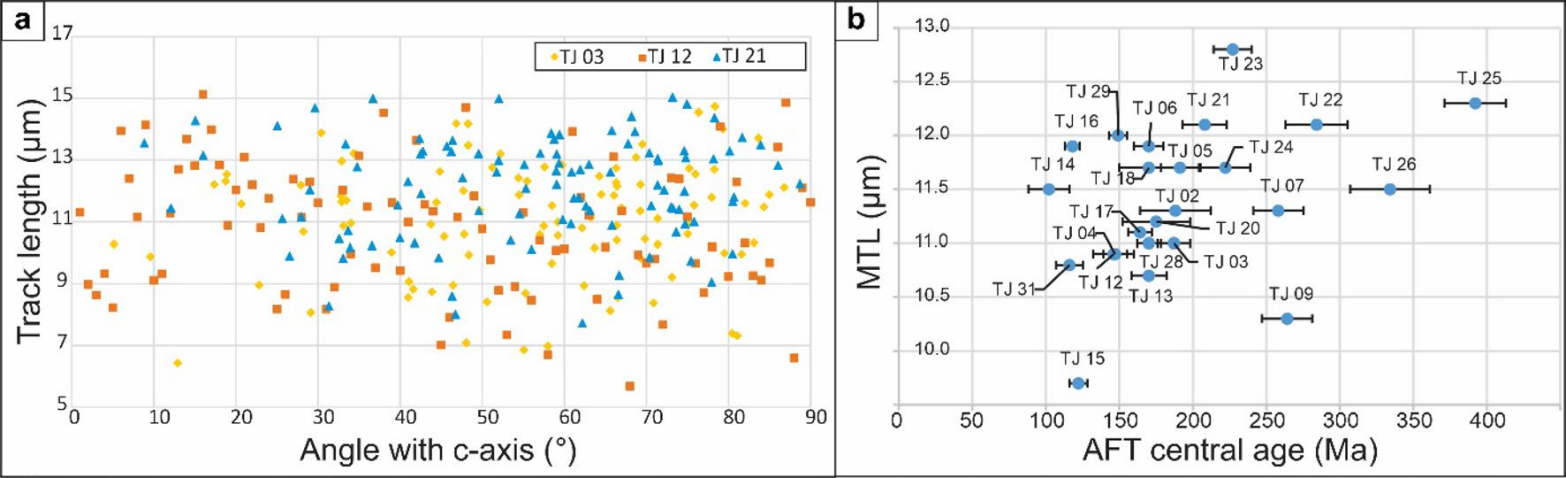
(**a**) Track length vs. angle with c-axis from confined tracks of samples TJ03, TJ12, and TJ21 (each representing one of the thermal history model groups: (i–iii). (**b**) MTL (μm) vs. AFT age (Ma) or “boomerang” plot^12^.

Despite the long-wavelength topography between 515 and 816 m elevation, and the low difference of erodibility of the sampled rocks (gneiss/migmatites), the AFT central ages vary substantially between 102 ± 14 Ma and 392 ± 21 Ma (Fig. 2), showing no correlation with D_par_ (see Supplementary S.7 online). The mean track length (MTL) values are short to intermediate (9.9 to 12.8 µm) with mainly unimodal distributions (see Supplementary S.1 online), indicating long residence in the apatite partial annealing zone (APAZ, c.120–60 °C). Track lengths and their angle with the c-axis show no evident correlation (see Supplementary S.8 and S.9 online). Neither is this the case for the MTL vs. AFT central age plot (Fig. 4). From the thermal history modelling, three main t–T trends can be distinguished (Fig. 2):

1. continuous, undisturbed, and slow cooling from the Paleozoic onwards; identified in the northernmost samples (TJ 21–26);
2. Paleozoic to Mesozoic fast to moderate cooling with subsequent, late c.50 °C cooling during the Eocene–present, as evidenced by samples from the Rio Pardo salient zone (TJ 09–16 and TJ 28–34); and
3. continuous and slow cooling from the Paleozoic to the Cenozoic, followed by c.35 °C of rapid cooling during the Eocene–present, as observed for the southernmost samples (TJ02–09) and samples geographically between the Rio Pardo salient and sample site TJ 21 (TJ17–20).

## Discussion

The AFT dataset distinctively reveals differential basement cooling and hence exhumation patterns. Samples from the northernmost area, close to Riacho de Santana city (TJ 21–26; models i in Fig. 2), do not show any important cooling during the Meso-Cenozoic. According to the models, slow and protracted cooling through the APAZ brought these samples (TJ 21–26) close to the surface (< 60 °C) even before the Jurassic, indicating that Cretaceous Atlantic rift and post-rift thermal events are lacking or exerted limited influence in this area. These samples (TJ 21–26) are from a previously identified cratonic segment of the Paramirim Aulacogen^22^, confirming no or very low degrees of tectonic rejuvenation of this lithospheric segment. The results are also in accordance with previous data from the southwestern SFC (Fig. 1), where AFT ages are older than the Jurassic^4^, and data from cratonic areas close to the Atlantic margin, where several samples are also older and do not seem affected by Cretaceous rifting or posterior events^23^ (Fig. 1). Areas with no clear signal in the AFT system from the rifting phase have indeed been associated to cratonic rheology (e.g.^4,24,25^). The rifting architecture varies greatly depending on the crustal thickness, the structural fabric and directions, the lithospheric rheology, and potential magmatic activity (e.g.^26–28^). With respect to our study region, we can conclude that the cratonic region of the Paramirim Aulacogen inherits the rigidity from the SFC. In this case, epirogenic uplift during the opening of the South Atlantic was mitigated as well as the erosional response to this process, resulting in an almost stable thermal structure.

Contrary to the craton, our data in the AB indicate relatively fast exhumation during the Eocene–present (models ii and iii in Fig. 2), with AFT ages not older than the Jurassic. Hence, confirming that our thermochronology data present a good correlation between the delimitation of the SFC and the AB. During the Cenozoic, the study area was embedded in the relatively stable South America platform, far from plate boundaries. The observed basement cooling must most probably be the consequence of erosional exhumation as indicated by the sediment supply to the adjacent basins. In the Eocene, the Jequitinhonha, Almada and Camamu passive margin basins, which are connected by river systems to our study area (Fig. 3), started their regressive phase. Progradation wedges, made up of coarse-grained sandstones, platform carbonates and distal mudstones, accumulated until the recent and reach 500 m in thickness^29–32^. Onshore, forming the typical regional tableland geomorphology, siliciclastic continental-to-shallow–water marine sediments of the Barreiras Formation were deposited from the Upper Oligocene onwards^33^. Thereby, we suggest that during the Eocene to present, our study area was heterogeneously eroded, likely partially contributing as a source of sediments to the above-mentioned deposits.

Previously published thermochronological data also indicates that the AB was indeed deeply exhumed during the Cenozoic (e.g.^4,14,23^) and, thus, our data further underscores the tracing of the belt (Fig. 2). Additionally, the Rio Pardo Salient seems one of the main structures in concentrating deformation since samples from this domain (TJ 11–16 and TJ 30–34) exhibit c.50 °C of rapid cooling during the Eocene to recent (models ii in Fig. 2). This salient presents a relatively high topography (c. 1000 m) in the Conquista Plateau region (Fig. 3), which may be a product of the reactivations from the Eocene. The formation of the Rio Pardo Salient resulted from the closure of the depocenter of the Neoproterozoic Macaúbas Basin, precursor to the AWCO collision^34^. The structure prior to the salient deformation was probably a major fault of the Macaúbas basin, deeply rooted through the crust. Analogue modelling has demonstrated that master faults are, indeed, more likely to concentrate stress and localize vertical displacements under compressional stress fields^35^. It is also important to note that the Rio Pardo Salient area (where the TJ 30–34 samples are located) limits the outcrops of the Macaúbas Basin fill (Fig. 2), supporting the location of the master fault.

Other authors also identified Neogene denudation pulses in northeastern Brazil using low-temperature thermochronology^36,37^, indicating that the event was widespread. Although climate has already been proposed as a main driving force for intensifying erosional denudation and hence basement cooling^37^, our results indicate that the denudation was far more heterogeneous. Evidence of brittle tectonics, related with ongoing ENE–WSW-oriented compression^38^, has been identified within the Cenozoic deposits (e.g.^39^) and challenged the concept of relative tectonic inactivity of passive margins. In line with the ideas of^36^, far-field partitioning of contemporaneous intraplate stress from the Andean collision zone seems to be the most plausible driving mechanism for the compressional tectonic reactivation of the intracontinental region, mainly during the Incaic and Quechua phases (Fig. 4). This final rapid cooling event most probably (partially) erased evidence from previous thermal events, including signals from West Gondwana break-up around 130 Ma. This latter event can however still be observed in three of our thermal history models (TJ 14–16).

**Figure 5:**
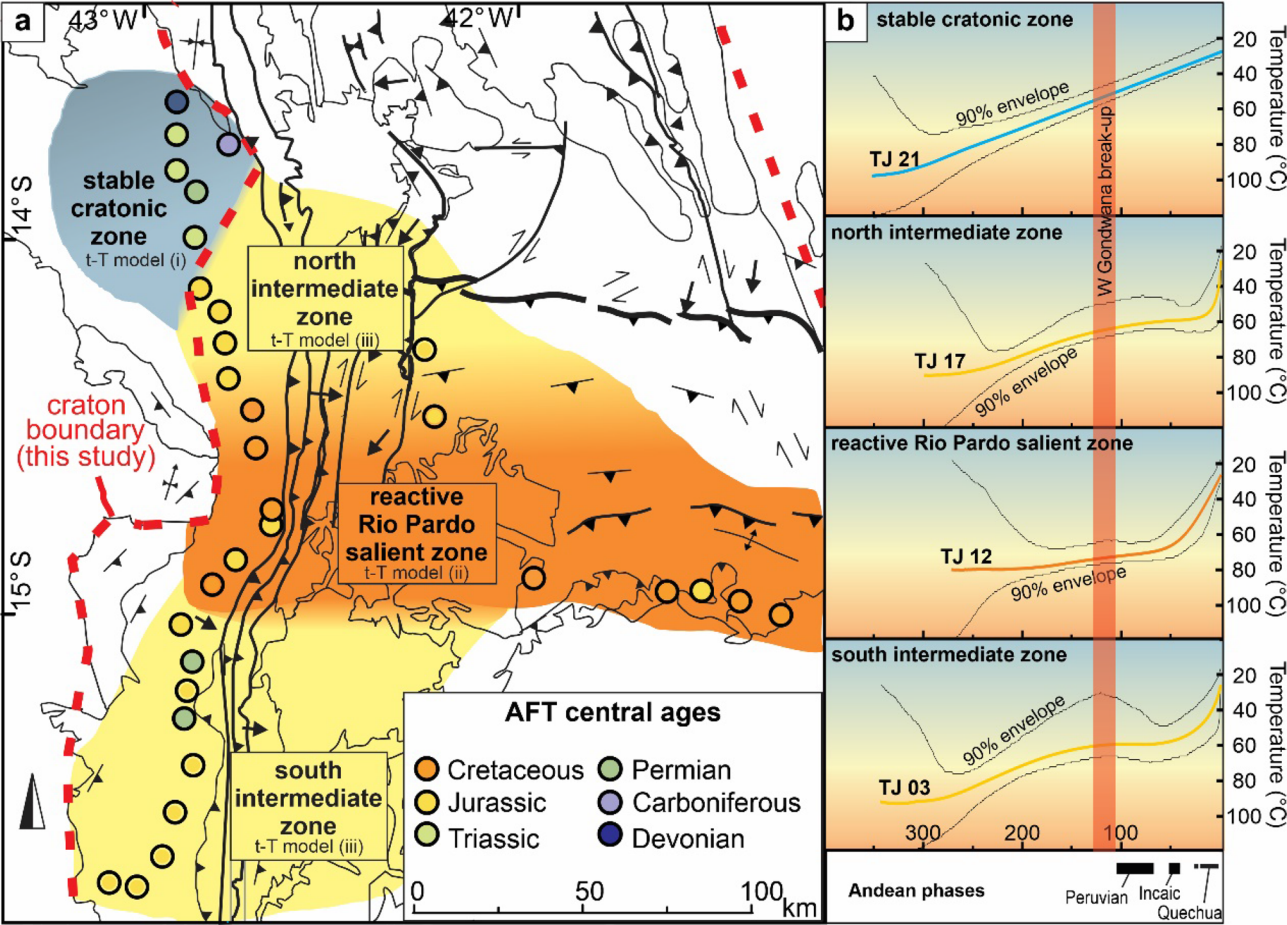
(**a**) AFT age distribution map of the study area identifying zones of tectonic reactivation with basement exhumation and stability. (**b**) Representative models for each zone and indication of the phases of Andean orogeny and West Gondwana break-up. The map was created using, ArcGIS 10.4.1 (https://www.esri.com) and Corel Draw Graphics Suite 2018 (http://www.coreldraw.com).

Although in some areas it is easy to distinguish craton vs. orogen behavior, the transition between them is not always clearly traceable. In general, stress and resulting deformation decrease from the collisional zone to the plate’s interior. In our data, we could identify an intermediate t–T path (models iii in Sect. 3; Fig. 2) with c.35 °C of Eocene–present cooling (from 60 °C to surface temperature). In this way, it is possible to use the decrease of recent basement cooling as documented by AFT data, to trace the transition from orogenic to cratonic lithosphere. In the main N–S transect (Fig. 2), this intermediate zone is about 40 km in S–N direction and was probably affected by AWCO orogeny but with less penetrative deformation. In the area where samples TJ 17–20 were collected, close to Guanambi city, a set of dextral reverse shear zones^40^ seems to be related to this intermediate zone and then part of the orogen area. Based on our observations, we propose an adjustment of the AB boundaries within the Paramirim Aulacogen (Fig. 2). We also suggest the compartmentalization of the orogen (Fig. 5) in weak zones (e.g. Rio Pardo Salient) and the area of decreasing rigidity (i.e. transitional zone) until finally the craton s.s. is reached. Remarkably, the heterogeneous basement cooling patterns in our study area, likely resulting from the same stress regime, reinforces the concept that some lithospheric segments are more easily deformed than others.

## Conclusions

AFT data from the São Francisco craton and adjoining Araçuaí belt in the Paramirim Aulacogen area (eastern Brazil) elucidate the differential behavior of the cratonic and non-cratonic lithosphere during the Phanerozoic exhumation of this region. To the north of our study area, thermal history modelling of the basement rocks exhibits slow and protracted cooling during the Phanerozoic, consistent with the rigid cratonic lithosphere of the São Francisco Craton. Samples from the Araçuaí belt, in the interior of the Paramirim aulacogen, display reactivation during the Cenozoic, mainly between the Eocene to present, reflecting its weakened lithosphere, inherited from the Ediacaran–Cambrian collision. An intermediate zone is identified, and it is considered mostly part of the Araçuaí Belt but with less penetrative deformation as to the orogen proper. The thermochronological data proved to be highly useful in determining the decreasing magnitude of reactivation along the craton—orogen boundary and can be used as a tool to trace and distinguish cratonic areas weakened by later deformation events.

## Supplementary Information

Supplementary Information.

## Data Availability

All data generated or analyzed during this study are included with the initial submission of the article in the form of Supplementary Information and are available on request.
