# Supplementary material for: Differential exhumation of cratonic and non-cratonic lithosphere revealed by apatite fission-track thermochronology along the edge of the São Francisco craton, eastern Brazil
Source: Sci Rep. 2022 Feb 17;12:2728. doi: 10.1038/s41598-022-06419-w (PMC8854403; doi:10.1038/s41598-022-06419-w)
Supplement: Supplementary file 1 — Supplementary Information. [file 41598_2022_6419_MOESM1_ESM.pdf]

Supplementary Information - supporting images, graphs, and table

S.1 - For each sample, the AFT age is displayed, together with its radial plots and data statistics. AFT c-axis corrected length data is displayed with each thermal history model. Results of inverse MCMC modelling in QTQt [1]. The expected model is displayed by the black curve, with the 90% credible interval. The colour scale indicates high probability in red and yellow, and the blue colours indicate lower probability values. The red line indicates the maximum likelihood model.

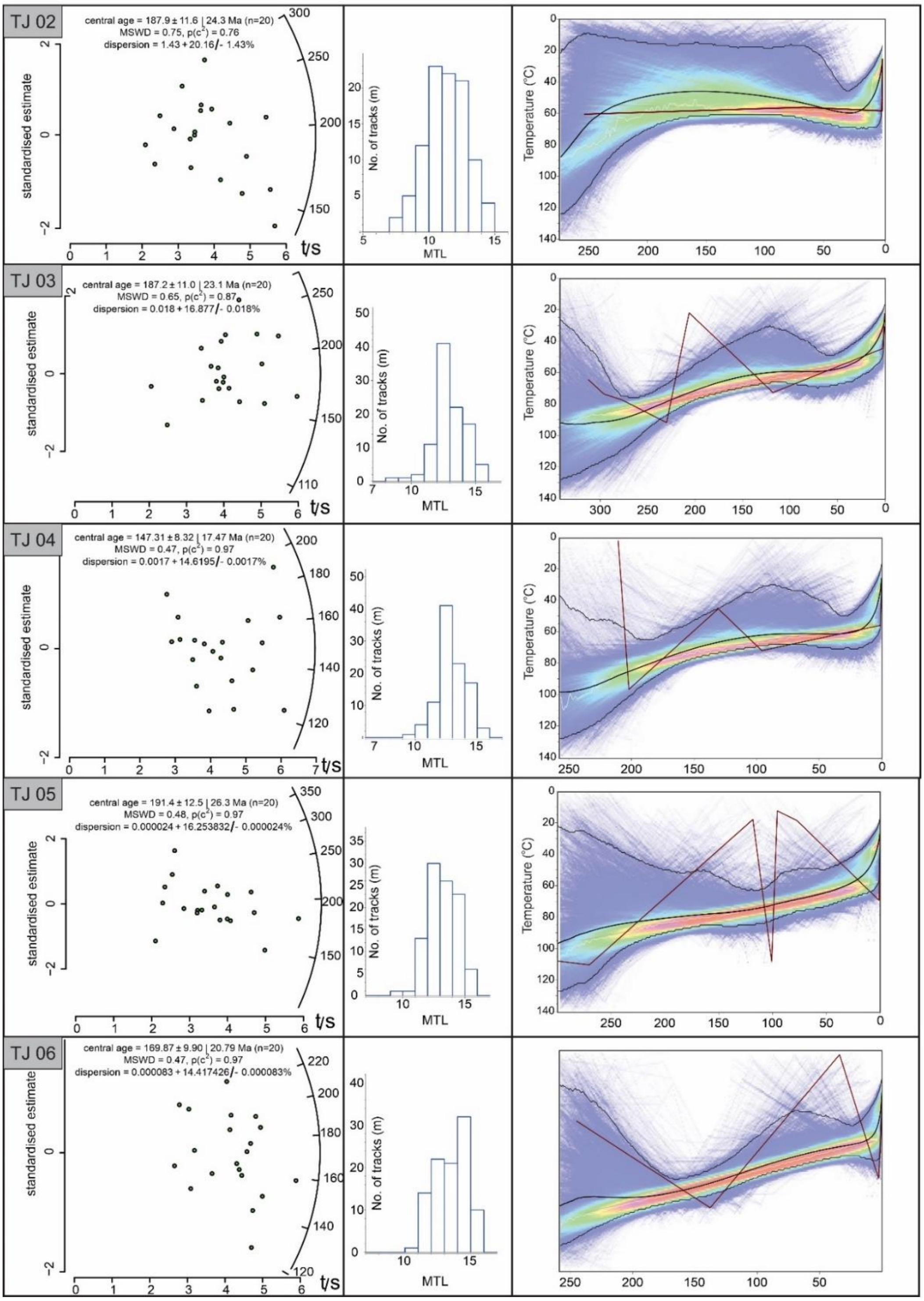

Fig.S1. continues on the next page

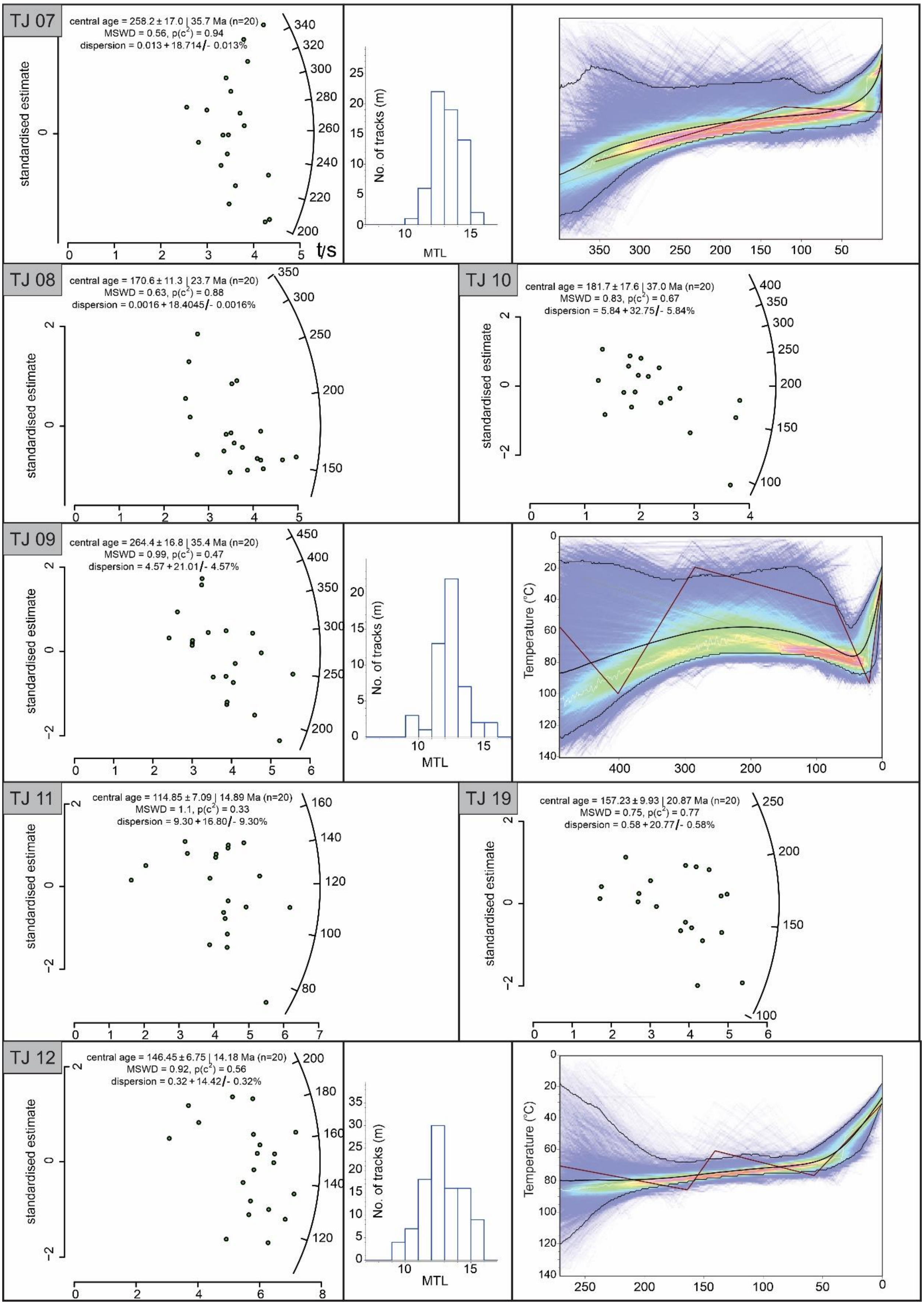

Fig.S1. continues on the next page

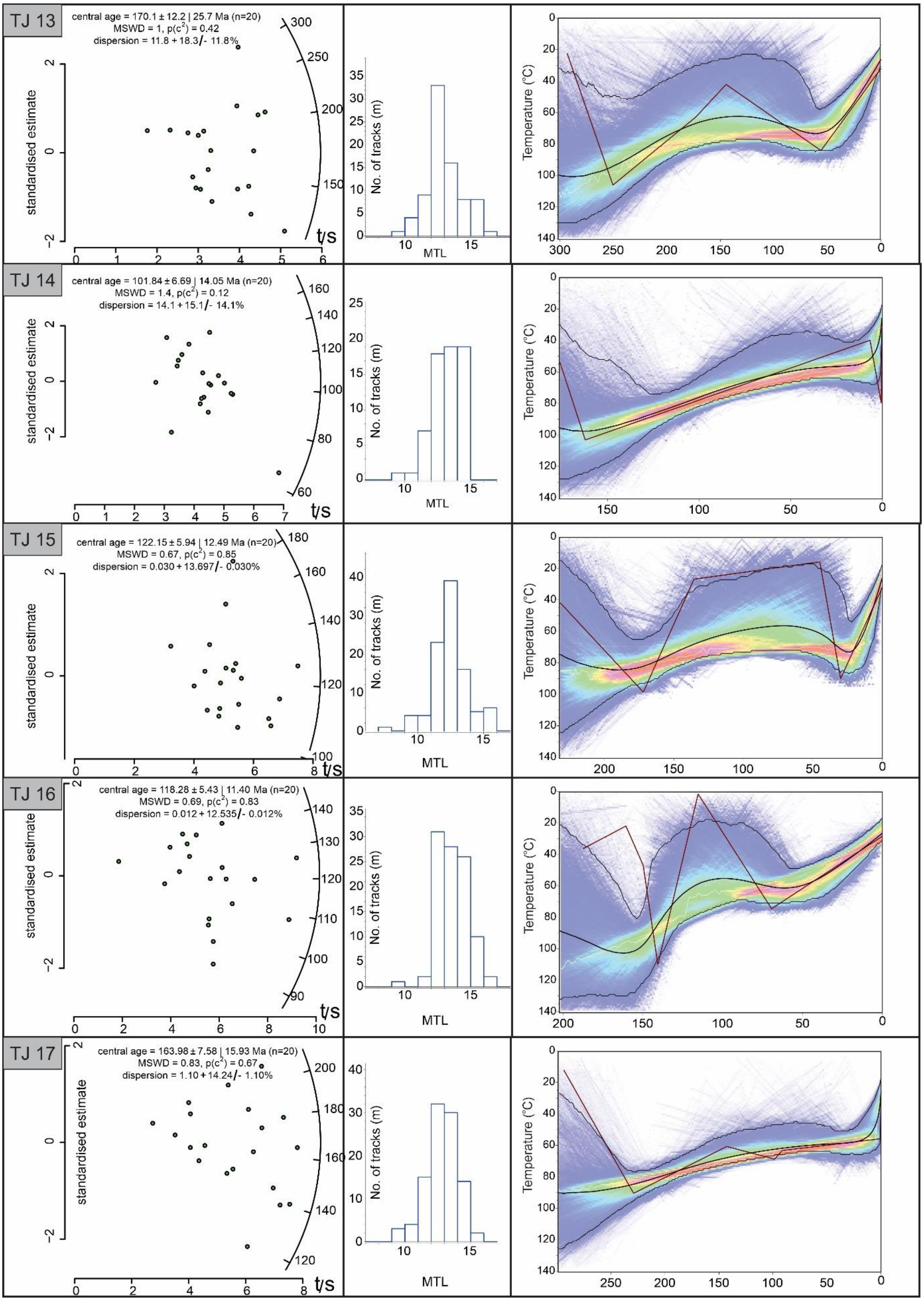

Fig.S1. continues on the next page

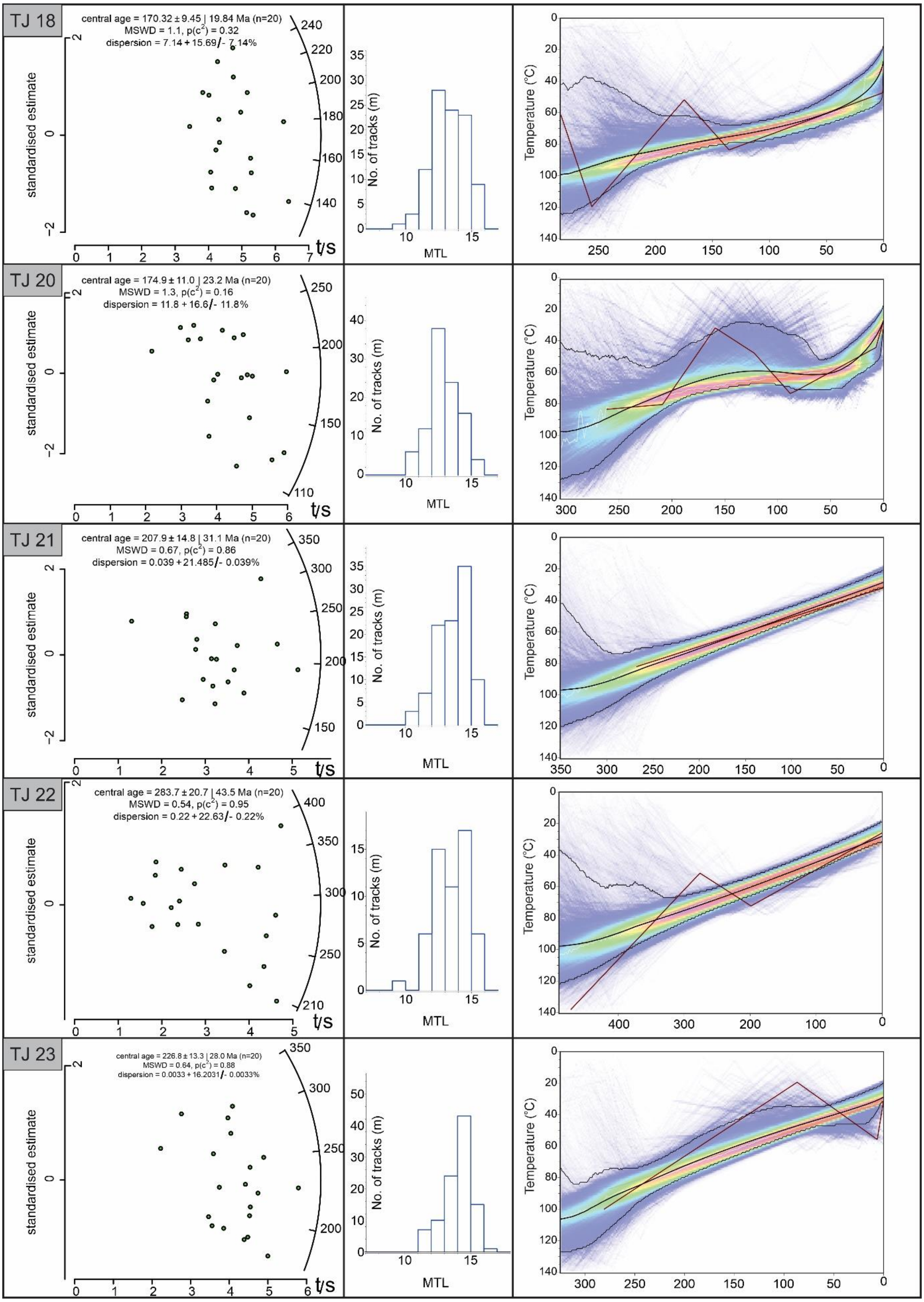

Fig.S1. continues on the next page

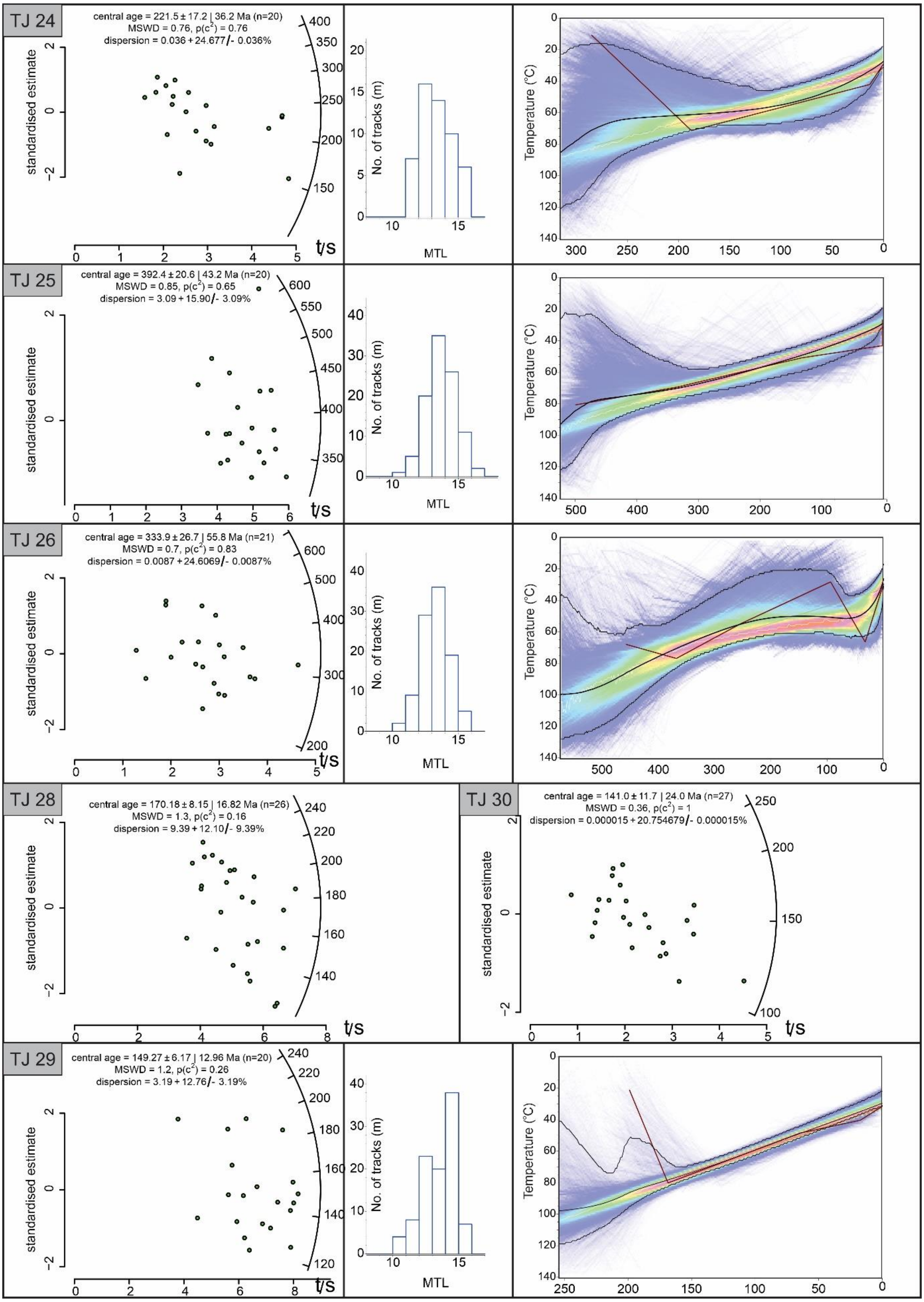

Fig.S1. continues on the next page

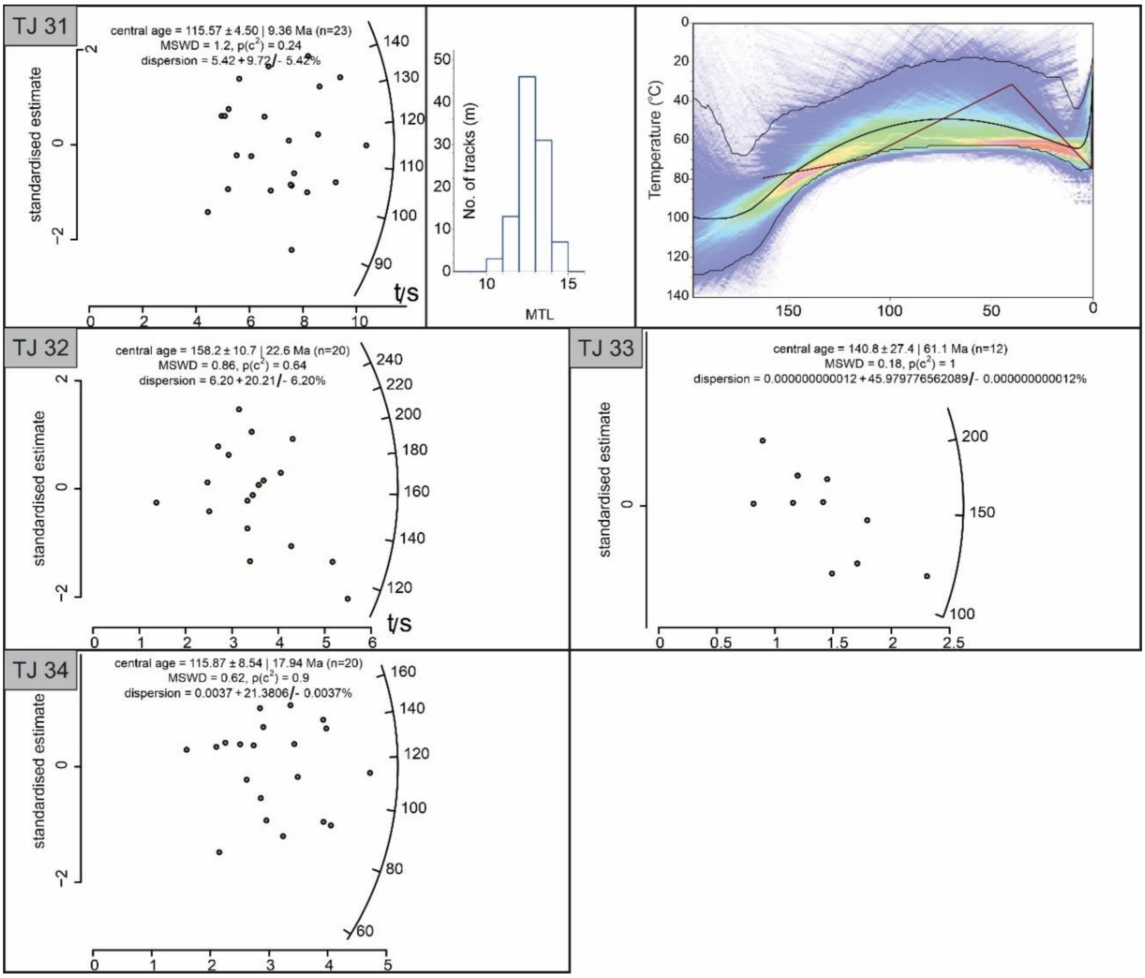

S.2 - Plot of Apatite Fission Track (AFT) central age vs. elevation.

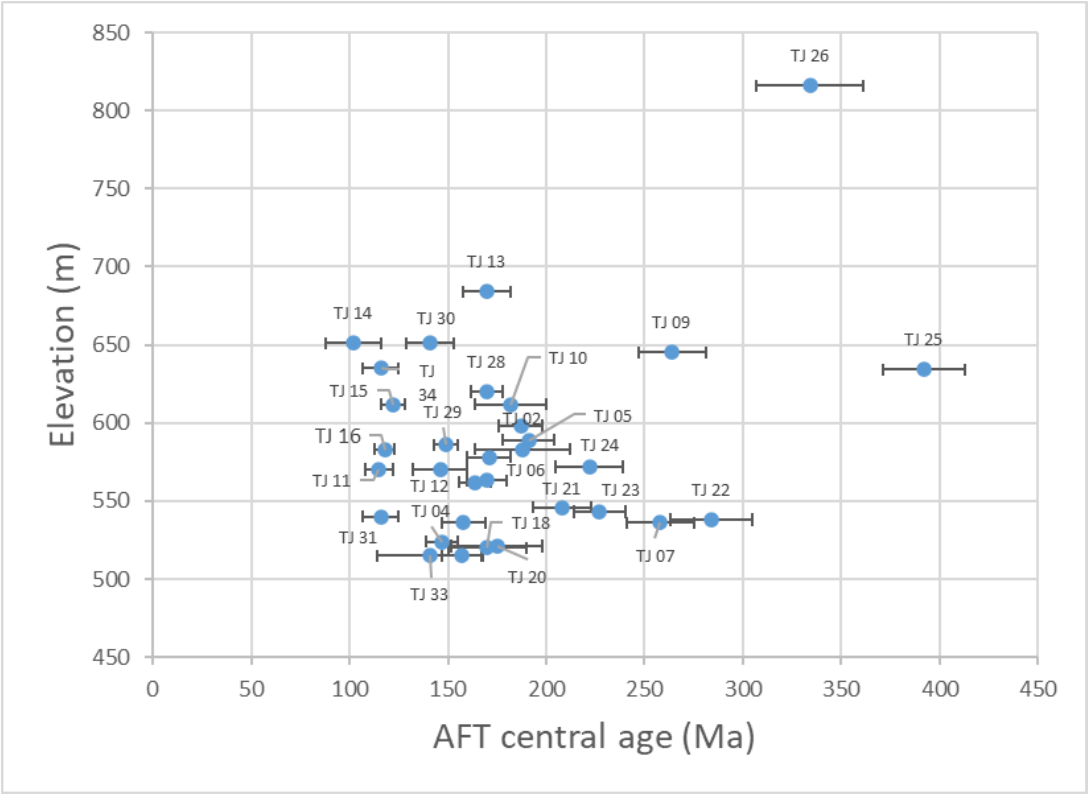

S.3 - Plot of mean track length (MTL, µm) vs. standard deviation (σ, in µm).

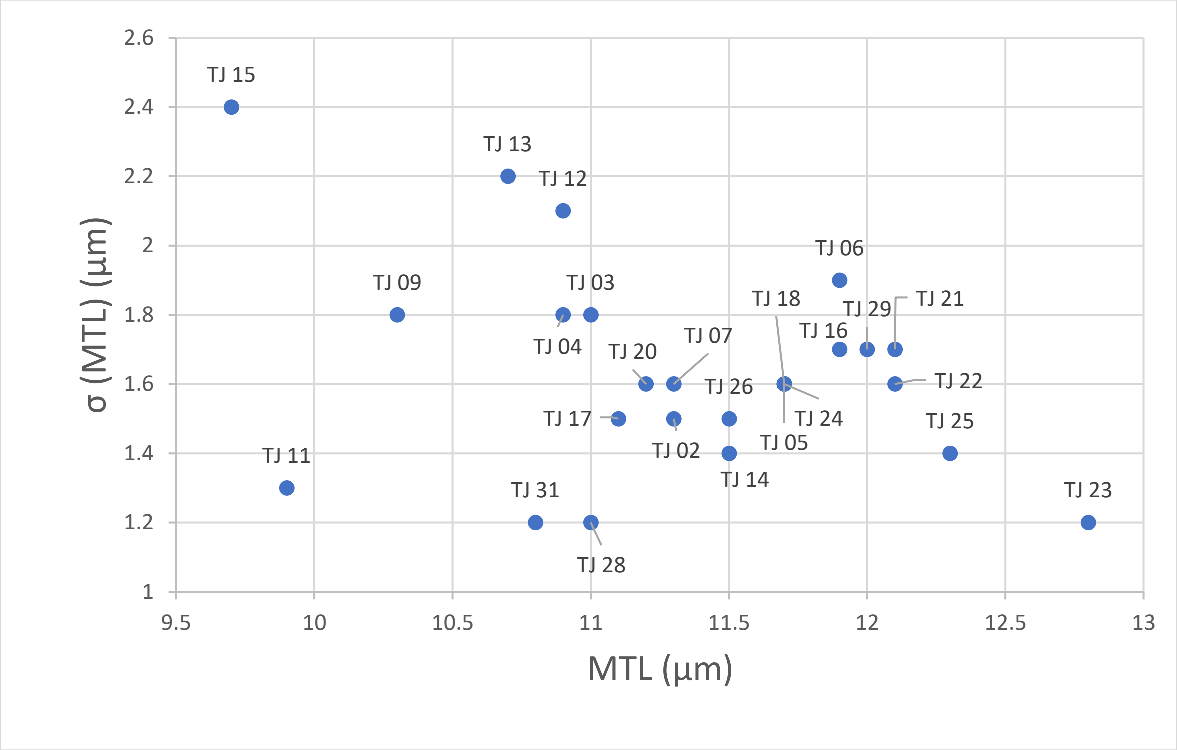

S.4 - Skewness plots vs. σ (MTL, in µm).

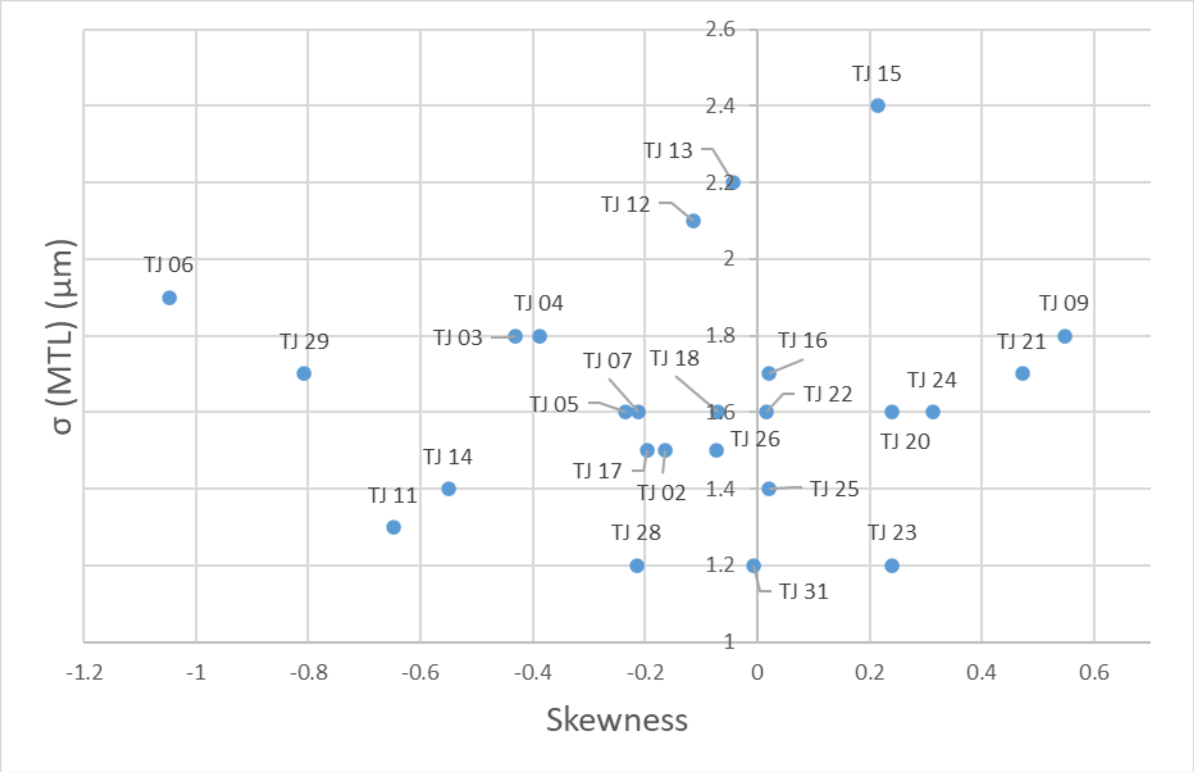

S.5 - Skewness plots vs. mean track length (MTL, µm).

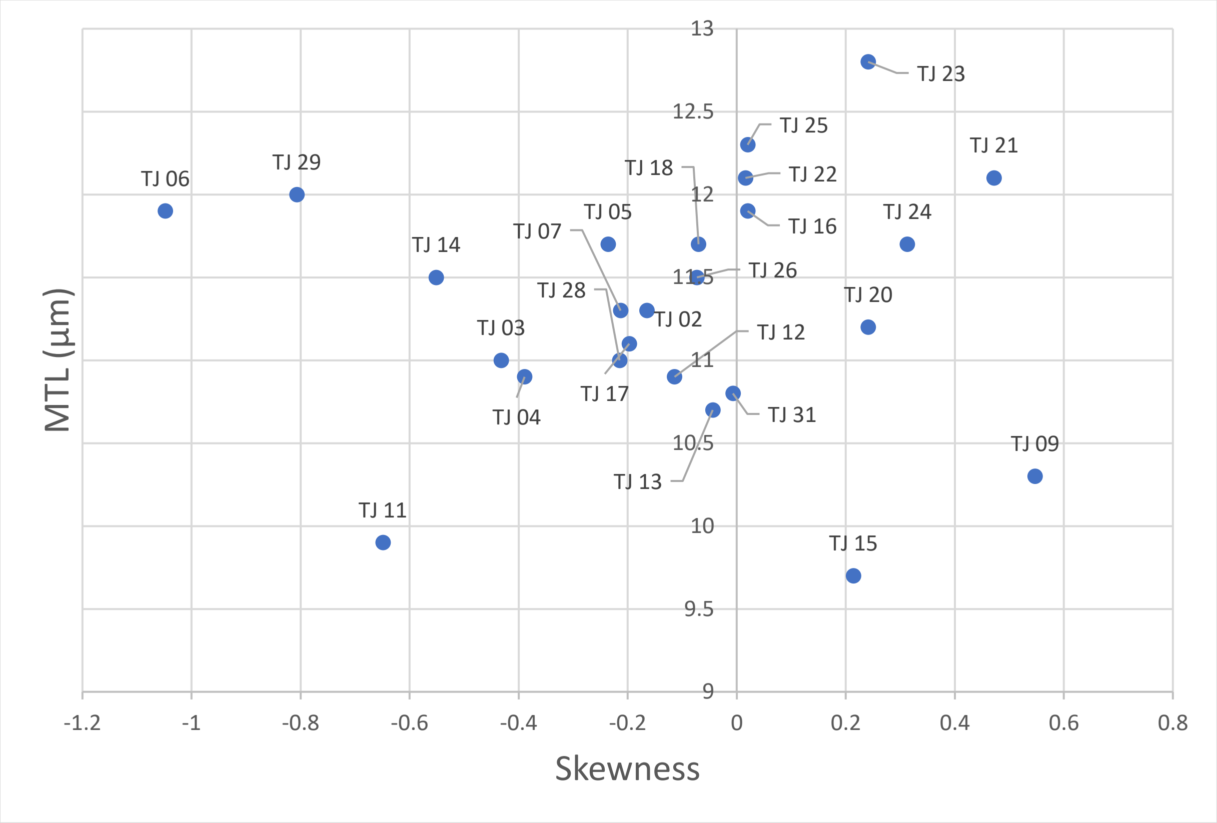

S.6 - AFT age vs. MTL (µm) or "boomerang" plot [2].

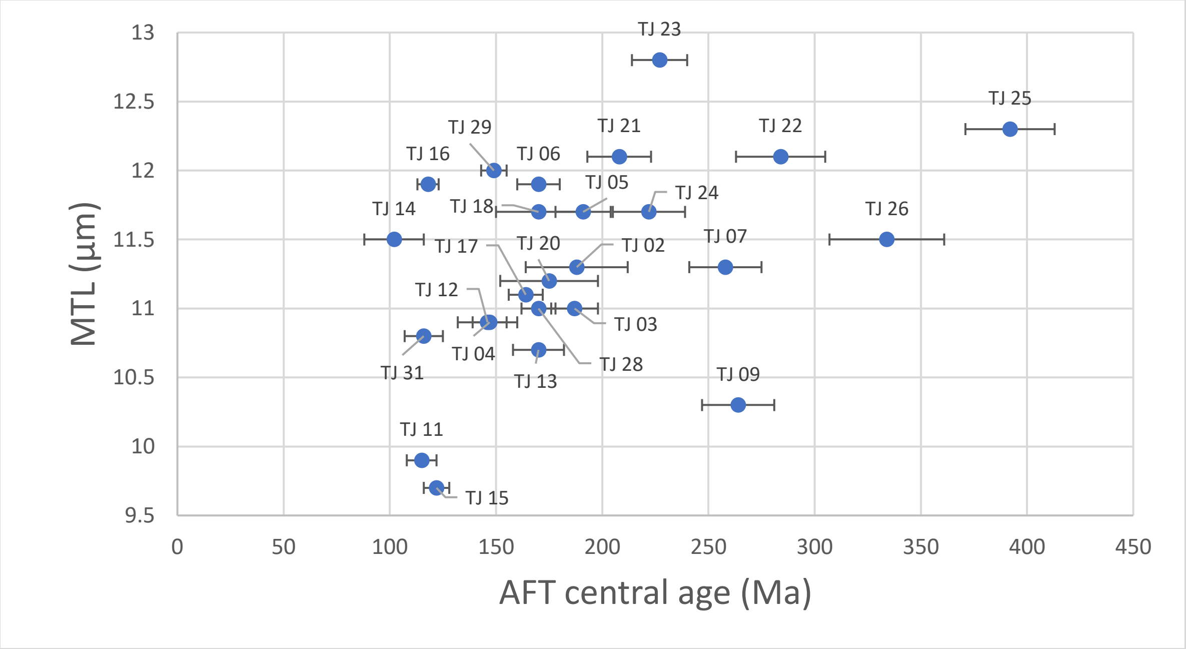

S.7 - AFT age (Ma) vs. D_{par} (µm) plot.

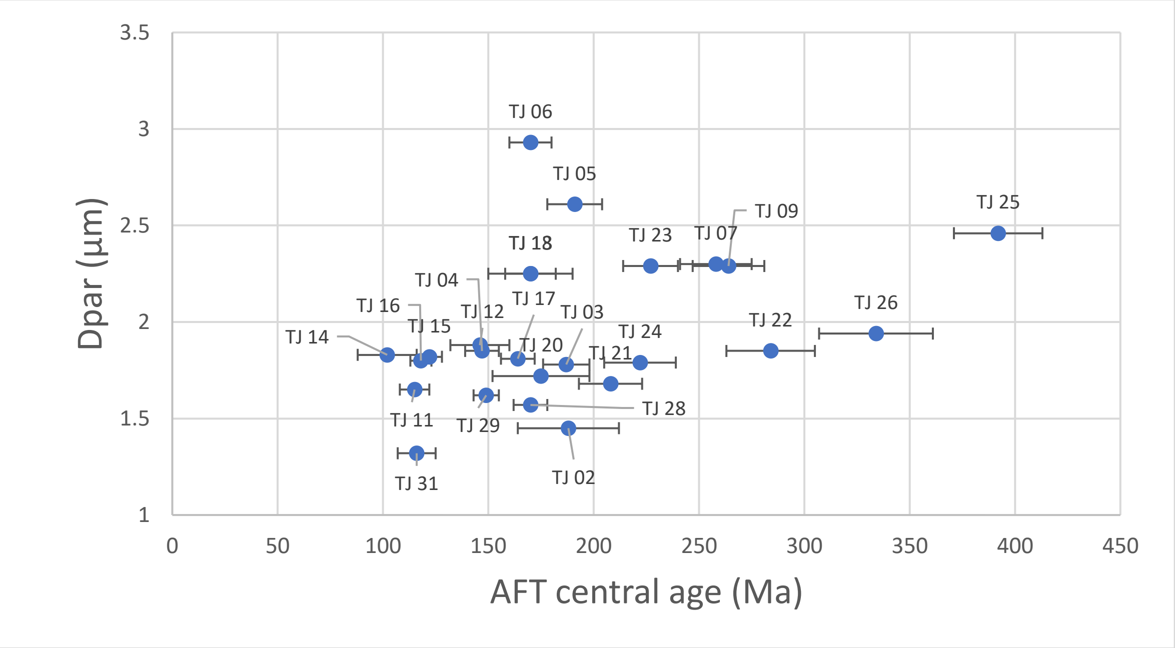

S.8 - Track length (µm) vs. angle with c-axis (°), n=2118. No clear trend can be deduced.

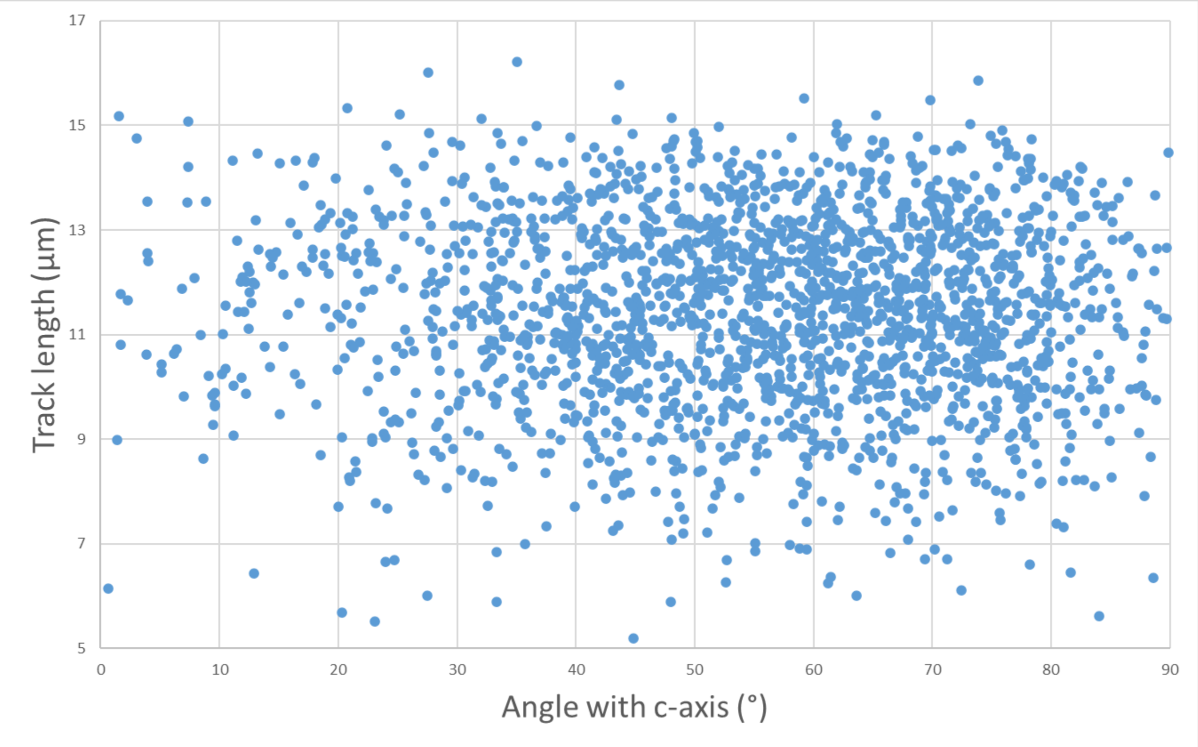

S.9 - Track length (μm) vs. angle with c-axis data (°) for each sample.

Sample	Track_ID	Length	C-axis angle
TJ 02	1	12.19	115.87
TJ 02	2	11.00	110.06
TJ 02	3	11.12	138.24
TJ 02	4	11.43	59.23
TJ 02	5	9.43	305.36
TJ 02	6	12.44	104.39
TJ 02	7	12.48	295.60
TJ 02	8	12.53	77.10
TJ 02	9	11.19	143.90
TJ 02	10	11.99	28.57
TJ 02	11	10.31	335.22
TJ 02	12	11.25	44.94
TJ 02	13	13.48	33.59
TJ 02	14	11.36	301.11
TJ 02	15	11.88	129.72
TJ 02	16	12.51	20.29
TJ 02	17	10.39	65.84
TJ 02	18	9.94	72.76
TJ 02	19	10.60	56.73
TJ 02	20	12.38	336.74
TJ 02	21	9.87	301.36
TJ 02	22	12.55	337.04
TJ 02	23	12.38	69.44
TJ 02	24	12.55	227.27
TJ 02	25	13.12	49.01
TJ 02	26	11.05	113.69
TJ 02	27	11.29	133.44
TJ 02	28	10.14	122.84
TJ 02	29	12.46	125.98
TJ 02	30	14.32	348.86
TJ 02	31	12.78	69.56
TJ 02	32	8.64	120.53
TJ 02	33	9.29	98.73
TJ 02	34	10.52	23.28
TJ 02	35	8.79	331.92
TJ 02	36	9.88	167.79
TJ 02	37	12.56	183.94
TJ 02	38	13.58	140.63
TJ 02	39	13.20	18.88
TJ 02	40	13.17	58.93
TJ 02	41	10.38	14.27
TJ 02	42	10.84	38.20
TJ 02	43	11.76	60.72

Sample	Track_ID	Length	C-axis angle
TJ 02	44	11.59	280.14
TJ 02	45	10.68	206.39
TJ 02	46	10.01	288.28
TJ 02	47	11.83	157.75
TJ 02	48	9.95	84.01
TJ 02	49	10.30	142.50
TJ 02	50	14.30	37.03
TJ 02	51	9.90	44.47
TJ 02	52	11.17	148.76
TJ 02	53	8.36	110.56
TJ 02	54	10.16	130.37
TJ 02	55	12.72	48.58
TJ 02	56	12.48	100.29
TJ 02	57	10.50	44.23
TJ 02	58	12.03	97.62
TJ 02	59	9.02	209.12
TJ 02	60	12.07	213.39
TJ 02	61	12.79	138.30
TJ 02	62	10.30	44.42
TJ 02	63	11.39	101.33
TJ 02	64	10.78	69.30
TJ 02	65	13.84	122.85
TJ 02	66	7.20	130.95
TJ 02	67	8.99	73.99
TJ 02	68	11.94	259.94
TJ 02	69	8.73	236.10
TJ 02	70	11.94	251.49
TJ 02	71	13.76	60.90
TJ 02	72	11.65	286.64
TJ 02	73	10.46	52.75
TJ 02	74	11.86	337.04
TJ 02	75	11.43	37.23
TJ 02	76	14.09	318.27
TJ 02	77	9.92	337.49
TJ 02	78	10.27	43.30
TJ 02	79	10.06	137.45
TJ 02	80	12.09	50.12
TJ 02	81	13.32	64.61
TJ 02	82	9.78	102.09
TJ 02	83	10.86	35.98
TJ 02	84	11.64	113.12
TJ 02	85	10.32	95.07
TJ 02	86	12.33	283.18

Sample	Track_ID	Length	C-axis angle
TJ 02	87	10.78	335.16
TJ 02	88	13.78	252.88
TJ 02	89	10.81	309.48
TJ 02	90	9.78	250.10
TJ 02	91	7.64	251.72
TJ 02	92	14.12	282.56
TJ 02	93	12.96	222.97
TJ 02	94	12.22	40.77
TJ 02	95	10.83	40.00
TJ 02	96	11.14	231.01
TJ 02	97	10.56	267.61
TJ 02	98	9.91	236.00
TJ 02	99	13.37	226.05
TJ 03	1	10.61	284.95
TJ 03	2	11.88	115.51
TJ 03	3	11.69	146.57
TJ 03	4	12.01	132.30
TJ 03	5	9.66	238.49
TJ 03	6	9.99	79.52
TJ 03	7	10.28	5.14
TJ 03	8	10.15	296.30
TJ 03	9	11.35	110.74
TJ 03	10	11.11	108.63
TJ 03	11	14.18	131.71
TJ 03	12	9.07	41.08
TJ 03	13	13.51	69.53
TJ 03	14	8.96	313.56
TJ 03	15	10.52	45.37
TJ 03	16	14.74	78.35
TJ 03	17	14.18	226.82
TJ 03	18	12.47	244.27
TJ 03	19	10.86	57.49
TJ 03	20	8.42	129.42
TJ 03	21	10.59	237.02
TJ 03	22	10.94	285.05
TJ 03	23	11.15	296.74
TJ 03	24	12.54	75.99
TJ 03	25	10.28	71.38
TJ 03	26	11.63	315.57
TJ 03	27	11.37	310.29
TJ 03	28	9.21	286.87
TJ 03	29	6.43	347.10
TJ 03	30	8.69	55.26
TJ 03	31	8.96	337.15
TJ 03	32	12.31	18.76
TJ 03	33	12.54	18.89
TJ 03	34	13.17	34.74
TJ 03	35	11.60	97.96
TJ 03	36	10.87	146.91
TJ 03	37	12.21	342.65
TJ 03	38	13.20	52.22
TJ 03	39	11.58	200.65
TJ 03	40	9.96	81.61
TJ 03	41	12.74	113.68
TJ 03	42	12.15	113.59
TJ 03	43	10.68	151.79
TJ 03	44	10.60	47.80
TJ 03	45	12.11	86.79
TJ 03	46	8.55	66.51
TJ 03	47	12.63	44.83
TJ 03	48	11.79	282.05
TJ 03	49	12.42	305.58
TJ 03	50	11.48	275.70
TJ 03	51	7.39	279.55
TJ 03	52	8.07	330.89
TJ 03	53	13.88	210.41
TJ 03	54	10.56	239.22
TJ 03	55	14.55	256.38
TJ 03	56	9.93	311.62
TJ 03	57	12.76	51.24
TJ 03	58	8.74	316.11
TJ 03	59	8.38	70.87
TJ 03	60	7.32	98.93
TJ 03	61	11.84	147.22
TJ 03	62	10.32	96.89
TJ 03	63	10.99	59.21
TJ 03	64	8.94	117.60
TJ 03	65	14.01	79.33
TJ 03	66	10.41	113.60
TJ 03	67	10.88	66.42
TJ 03	68	10.92	43.75
TJ 03	69	12.19	27.92
TJ 03	70	12.70	78.28
TJ 03	71	10.80	297.85
TJ 03	72	10.02	321.04
TJ 03	73	11.87	125.51
TJ 03	74	6.86	55.07
TJ 03	75	13.09	68.75
TJ 03	76	9.88	170.42
TJ 03	77	12.77	122.41

Sample	Track_ID	Length	C-axis angle
TJ 03	78	11.54	75.81
TJ 03	79	12.21	249.29
TJ 03	80	10.44	110.15
TJ 03	81	7.09	131.93
TJ 03	82	8.82	138.40
TJ 03	83	9.82	69.28
TJ 03	84	13.21	145.65
TJ 03	85	12.97	327.12
TJ 03	86	11.85	246.05
TJ 03	87	8.55	319.00
TJ 03	88	11.65	327.12
TJ 03	89	6.98	238.01
TJ 03	90	11.45	293.04
TJ 03	91	11.52	301.48
TJ 03	92	11.88	275.03
TJ 03	93	13.71	276.33
TJ 03	94	8.13	245.60
TJ 03	95	10.96	214.01
TJ 03	96	12.73	284.78
TJ 03	97	12.32	235.45
TJ 03	98	12.27	246.33
TJ 03	99	13.48	311.70
TJ 03	100	12.84	239.73
TJ 04	1	9.19	257.89
TJ 04	2	6.70	249.36
TJ 04	3	12.54	306.97
TJ 04	4	10.25	217.36
TJ 04	5	11.95	245.28
TJ 04	6	11.50	296.03
TJ 04	7	9.35	51.26
TJ 04	8	7.67	308.50
TJ 04	9	11.49	96.67
TJ 04	10	11.16	40.21
TJ 04	11	12.78	66.34
TJ 04	12	12.61	64.74
TJ 04	13	12.25	289.68
TJ 04	14	9.40	300.71
TJ 04	15	10.25	229.40
TJ 04	16	11.45	247.43
TJ 04	17	11.14	286.02
TJ 04	18	10.81	218.88
TJ 04	19	9.03	229.12
TJ 04	20	11.67	251.96
TJ 04	21	10.41	280.66
TJ 04	22	8.57	278.79

Sample	Track_ID	Length	C-axis angle
TJ 04	23	11.36	213.48
TJ 04	24	10.81	29.67
TJ 04	25	10.07	311.45
TJ 04	26	11.90	299.84
TJ 04	27	10.68	254.44
TJ 04	28	13.54	208.96
TJ 04	29	12.83	252.50
TJ 04	30	13.64	216.36
TJ 04	31	9.92	329.45
TJ 04	32	9.47	320.04
TJ 04	33	8.78	294.24
TJ 04	34	10.04	294.07
TJ 04	35	12.06	284.43
TJ 04	36	12.79	232.04
TJ 04	37	15.02	242.00
TJ 04	38	12.02	299.05
TJ 04	39	9.96	258.00
TJ 04	40	13.20	218.29
TJ 04	41	11.52	289.24
TJ 04	42	9.63	74.25
TJ 04	43	10.91	315.70
TJ 04	44	9.35	282.24
TJ 04	45	12.71	307.09
TJ 04	46	10.74	237.45
TJ 04	47	12.46	293.39
TJ 04	48	11.92	291.11
TJ 04	49	9.82	303.57
TJ 04	50	9.55	222.43
TJ 04	51	11.98	308.79
TJ 04	52	12.96	47.25
TJ 04	53	10.36	305.97
TJ 04	54	7.33	37.54
TJ 04	55	11.22	314.43
TJ 04	56	12.52	246.81
TJ 04	57	10.85	305.30
TJ 04	58	8.09	232.16
TJ 04	59	11.82	296.67
TJ 04	60	9.96	295.94
TJ 04	61	11.49	284.47
TJ 04	62	13.28	204.52
TJ 04	63	11.28	71.95
TJ 04	64	13.61	226.11
TJ 04	65	11.10	33.95
TJ 04	66	4.87	227.89
TJ 04	67	8.41	210.34

Differential exhumation of cratonic and non-cratonic lithosphere revealed by apatite fission track thermochronology along the Paramirim aulacogen, Brazil --- Authors: Ana Fonseca, Simone Cruz, Tiago Novo, Zhiyuan He, Johan De Grave

Sample	Track_ID	Length	C-axis angle
TJ 04	68	7.94	231.72
TJ 04	69	12.04	229.67
TJ 04	70	9.73	232.77
TJ 04	71	12.87	250.00
TJ 04	72	9.21	121.00
TJ 04	73	10.06	343.18
TJ 04	74	10.62	264.00
TJ 04	75	10.18	233.51
TJ 04	76	8.62	256.99
TJ 04	77	11.60	318.13
TJ 04	78	9.81	281.66
TJ 04	79	10.20	336.59
TJ 04	80	10.06	299.43
TJ 04	81	9.58	233.78
TJ 04	82	11.45	295.43
TJ 04	83	14.40	40.86
TJ 04	84	13.82	279.09
TJ 04	85	11.22	314.49
TJ 04	86	13.60	303.28
TJ 04	87	10.80	235.87
TJ 04	88	9.02	337.14
TJ 04	89	12.49	305.31
TJ 04	90	10.36	35.34
TJ 04	91	11.94	32.29
TJ 04	92	11.04	273.91
TJ 04	93	8.85	244.38
TJ 04	94	12.31	327.15
TJ 04	95	11.66	247.21
TJ 04	96	12.24	252.94
TJ 04	97	8.31	223.77
TJ 04	98	10.04	256.53
TJ 04	99	12.88	266.52
TJ 04	100	9.52	203.84
TJ 05	1	11.55	104.43
TJ 05	2	11.56	50.53
TJ 05	3	12.85	319.52
TJ 05	4	9.68	39.05
TJ 05	5	10.73	44.33
TJ 05	6	12.05	304.30
TJ 05	7	10.44	50.27
TJ 05	8	11.28	326.60
TJ 05	9	12.92	339.39
TJ 05	10	11.39	277.12
TJ 05	11	12.01	191.76
TJ 05	12	11.13	315.40

Sample	Track_ID	Length	C-axis angle
TJ 05	13	11.21	230.24
TJ 05	14	11.48	268.90
TJ 05	15	13.68	306.51
TJ 05	16	11.77	332.74
TJ 05	17	13.46	265.20
TJ 05	18	13.28	264.44
TJ 05	19	12.39	230.32
TJ 05	20	9.96	237.86
TJ 05	21	12.67	269.71
TJ 05	22	9.97	314.70
TJ 05	23	10.97	44.60
TJ 05	24	14.51	43.60
TJ 05	25	12.48	339.50
TJ 05	26	11.25	47.09
TJ 05	27	10.46	42.44
TJ 05	28	11.69	281.35
TJ 05	29	12.23	55.01
TJ 05	30	14.45	244.78
TJ 05	31	12.53	276.65
TJ 05	32	7.80	293.16
TJ 05	33	11.18	235.82
TJ 05	34	14.10	244.77
TJ 05	35	9.96	303.73
TJ 05	36	11.72	54.68
TJ 05	37	13.23	326.18
TJ 05	38	15.20	65.26
TJ 05	39	11.37	286.44
TJ 05	40	11.70	50.15
TJ 05	41	11.61	196.70
TJ 05	42	11.99	224.46
TJ 05	43	9.19	248.49
TJ 05	44	10.17	236.04
TJ 05	45	14.43	283.67
TJ 05	46	10.50	309.69
TJ 05	47	11.57	302.26
TJ 05	48	12.64	88.91
TJ 05	49	12.93	58.76
TJ 05	50	9.66	329.91
TJ 05	51	9.34	52.43
TJ 05	52	9.30	47.02
TJ 05	53	9.10	66.78
TJ 05	54	13.25	203.47
TJ 05	55	8.45	243.33
TJ 05	56	10.41	240.83
TJ 05	57	13.56	217.11

Differential exhumation of cratonic and non-cratonic lithosphere revealed by apatite fission track thermochronology along the Paramirim aulacogen, Brazil --- Authors: Ana Fonseca, Simone Cruz, Tiago Novo, Zhiyuan He, Johan De Grave

Sample	Track_ID	Length	C-axis angle
TJ 05	58	12.79	297.10
TJ 05	59	10.81	313.55
TJ 05	60	12.81	323.47
TJ 05	61	10.97	140.60
TJ 05	62	8.15	79.00
TJ 05	63	11.71	39.00
TJ 05	64	13.82	33.90
TJ 05	65	13.08	29.71
TJ 05	66	13.07	120.13
TJ 05	67	9.39	115.32
TJ 05	68	12.28	29.20
TJ 05	69	13.96	126.63
TJ 05	70	10.43	39.67
TJ 05	71	12.57	62.64
TJ 05	72	13.77	125.87
TJ 05	73	11.58	102.92
TJ 05	74	9.41	48.65
TJ 05	75	11.73	114.49
TJ 05	76	12.81	78.84
TJ 05	77	10.37	311.04
TJ 05	78	14.64	294.03
TJ 05	79	9.64	9.64
TJ 05	80	12.24	251.82
TJ 05	81	9.93	299.22
TJ 05	82	11.73	297.98
TJ 05	83	11.90	300.36
TJ 05	84	9.90	43.82
TJ 05	85	13.21	44.19
TJ 05	86	13.31	299.51
TJ 05	87	10.53	124.21
TJ 05	88	11.57	290.24
TJ 05	89	12.20	167.46
TJ 05	90	13.23	315.97
TJ 05	91	9.69	240.67
TJ 05	92	11.37	281.52
TJ 05	93	13.36	320.69
TJ 05	94	10.98	105.43
TJ 05	95	13.23	51.14
TJ 05	96	7.52	70.55
TJ 05	97	11.44	11.55
TJ 05	98	12.65	87.32
TJ 05	99	13.63	252.11
TJ 05	100	9.64	53.28
TJ 06	1	10.19	261.68
TJ 06	2	4.94	240.33

Sample	Track_ID	Length	C-axis angle
TJ 06	3	11.35	294.19
TJ 06	4	13.27	218.85
TJ 06	5	13.67	268.77
TJ 06	6	13.11	298.55
TJ 06	7	10.49	117.94
TJ 06	8	8.21	81.01
TJ 06	9	9.40	320.94
TJ 06	10	11.31	69.95
TJ 06	11	13.31	305.20
TJ 06	12	9.79	60.51
TJ 06	13	13.53	299.12
TJ 06	14	10.99	246.76
TJ 06	15	12.84	63.31
TJ 06	16	11.89	32.52
TJ 06	17	12.75	72.18
TJ 06	18	13.32	20.76
TJ 06	19	11.21	21.16
TJ 06	20	11.36	325.28
TJ 06	21	12.37	55.14
TJ 06	22	11.77	77.54
TJ 06	23	12.53	72.45
TJ 06	24	12.71	59.63
TJ 06	25	11.74	124.88
TJ 06	26	11.15	304.45
TJ 06	27	10.13	263.51
TJ 06	28	13.61	316.78
TJ 06	29	13.28	262.83
TJ 06	30	13.45	264.66
TJ 06	31	9.63	251.79
TJ 06	32	10.23	315.99
TJ 06	33	12.04	260.08
TJ 06	34	10.52	296.07
TJ 06	35	12.29	262.38
TJ 06	36	13.77	22.54
TJ 06	37	11.04	70.02
TJ 06	38	10.75	243.15
TJ 06	39	10.83	225.80
TJ 06	40	12.98	180.66
TJ 06	41	12.64	234.93
TJ 06	42	8.72	325.82
TJ 06	43	11.39	319.37
TJ 06	44	10.03	308.51
TJ 06	45	13.03	257.97
TJ 06	46	15.15	228.09
TJ 06	47	5.51	203.09

Differential exhumation of cratonic and non-cratonic lithosphere revealed by apatite fission track thermochronology along the Paramirim aulacogen, Brazil --- Authors: Ana Fonseca, Simone Cruz, Tiago Novo, Zhiyuan He, Johan De Grave

Sample	Track_ID	Length	C-axis angle
TJ 06	48	12.82	236.43
TJ 06	49	9.03	41.01
TJ 06	50	14.68	50.08
TJ 06	51	14.19	111.06
TJ 06	52	9.54	131.87
TJ 06	53	11.77	358.30
TJ 06	54	12.24	39.51
TJ 06	55	12.31	167.59
TJ 06	56	11.63	319.04
TJ 06	57	11.13	255.30
TJ 06	58	12.62	238.17
TJ 06	59	9.74	316.32
TJ 06	60	12.68	42.39
TJ 06	61	14.17	235.34
TJ 06	62	14.00	30.61
TJ 06	63	10.16	293.69
TJ 06	64	12.56	295.75
TJ 06	65	13.69	318.80
TJ 06	66	10.88	324.17
TJ 06	67	12.35	299.84
TJ 06	68	13.88	43.31
TJ 06	69	13.87	234.00
TJ 06	70	10.98	241.93
TJ 06	71	13.82	326.05
TJ 06	72	11.61	274.52
TJ 06	73	13.27	67.35
TJ 06	74	9.19	120.67
TJ 06	75	14.18	327.19
TJ 06	76	11.13	85.61
TJ 06	77	11.57	70.56
TJ 06	78	13.08	60.00
TJ 06	79	14.18	24.74
TJ 06	80	11.56	298.12
TJ 06	81	10.88	106.85
TJ 06	82	14.37	47.89
TJ 06	83	14.03	55.56
TJ 06	84	11.27	283.40
TJ 06	85	13.14	303.19
TJ 06	86	13.69	318.17
TJ 06	87	9.78	238.89
TJ 06	88	13.73	308.86
TJ 06	89	14.33	16.40
TJ 06	90	10.15	221.43
TJ 06	91	11.90	300.31
TJ 06	92	11.82	295.99

Sample	Track_ID	Length	C-axis angle
TJ 06	93	12.27	240.68
TJ 06	94	12.54	345.72
TJ 06	95	8.22	262.79
TJ 06	96	14.57	227.59
TJ 06	97	12.95	325.99
TJ 06	98	13.87	260.33
TJ 06	99	11.38	340.06
TJ 06	100	13.02	237.34
TJ 07	1	10.91	81.61
TJ 07	2	8.64	65.03
TJ 07	3	8.36	74.12
TJ 07	4	13.63	128.23
TJ 07	5	13.07	210.52
TJ 07	6	10.83	282.65
TJ 07	7	10.08	320.77
TJ 07	8	11.51	61.91
TJ 07	9	9.84	88.00
TJ 07	10	10.99	72.62
TJ 07	11	12.97	80.00
TJ 07	12	13.16	85.20
TJ 07	13	11.02	52.05
TJ 07	14	11.79	58.38
TJ 07	15	10.23	245.18
TJ 07	16	12.77	235.69
TJ 07	17	12.17	296.17
TJ 07	18	13.13	284.69
TJ 07	19	10.18	252.93
TJ 07	20	13.06	292.72
TJ 07	21	10.38	32.38
TJ 07	22	11.79	284.99
TJ 07	23	12.50	39.27
TJ 07	24	13.13	237.99
TJ 07	25	14.06	81.37
TJ 07	26	10.72	32.41
TJ 07	27	12.99	51.93
TJ 07	28	10.01	303.07
TJ 07	29	10.44	73.66
TJ 07	30	11.52	69.21
TJ 07	31	9.55	132.50
TJ 07	32	10.58	42.55
TJ 07	33	11.14	301.19
TJ 07	34	12.22	74.05
TJ 07	35	12.07	24.45
TJ 07	36	8.74	37.84
TJ 07	37	9.77	33.31

Differential exhumation of cratonic and non-cratonic lithosphere revealed by apatite fission track thermochronology along the Paramirim aulacogen, Brazil --- Authors: Ana Fonseca, Simone Cruz, Tiago Novo, Zhiyuan He, Johan De Grave

Sample	Track_ID	Length	C-axis angle
TJ 07	38	12.31	284.01
TJ 07	39	9.83	113.51
TJ 07	40	8.40	228.28
TJ 07	41	11.28	256.28
TJ 07	42	12.07	285.55
TJ 07	43	8.34	328.52
TJ 07	44	13.06	328.26
TJ 07	45	12.80	348.52
TJ 07	46	12.63	346.69
TJ 07	47	11.17	287.37
TJ 07	48	9.26	257.61
TJ 07	49	13.56	278.05
TJ 07	50	12.14	241.94
TJ 07	51	8.60	307.53
TJ 07	52	10.06	294.64
TJ 07	53	14.17	82.64
TJ 07	54	8.84	50.57
TJ 07	55	13.53	187.29
TJ 07	56	11.75	235.80
TJ 07	57	11.29	229.67
TJ 07	58	9.56	225.42
TJ 07	59	12.72	262.94
TJ 07	60	11.07	254.40
TJ 07	61	11.73	238.89
TJ 07	62	11.38	250.07
TJ 07	63	9.49	237.91
TJ 07	64	13.74	226.85
TJ 07	65	12.51	76.73
TJ 09	1	11.09	300.89
TJ 09	2	9.84	294.08
TJ 09	3	9.09	310.02
TJ 09	4	10.58	74.75
TJ 09	5	11.00	71.46
TJ 09	6	8.74	37.16
TJ 09	7	10.85	21.82
TJ 09	8	10.73	230.52
TJ 09	9	14.27	281.86
TJ 09	10	11.72	322.08
TJ 09	11	11.87	304.33
TJ 09	12	9.82	252.25
TJ 09	13	10.94	247.30
TJ 09	14	10.45	304.65
TJ 09	15	8.18	110.65
TJ 09	16	11.27	207.58
TJ 09	17	9.40	35.32

Sample	Track_ID	Length	C-axis angle
TJ 09	18	8.44	49.00
TJ 09	19	9.11	229.98
TJ 09	20	9.58	50.67
TJ 09	21	15.18	358.44
TJ 09	22	10.00	102.78
TJ 09	23	7.82	119.28
TJ 09	24	6.69	335.29
TJ 09	25	8.66	268.37
TJ 09	26	12.14	56.54
TJ 09	27	8.91	50.47
TJ 09	28	12.92	163.43
TJ 09	29	7.89	53.73
TJ 09	30	11.74	311.25
TJ 09	31	13.57	238.65
TJ 09	32	8.58	311.56
TJ 09	33	9.95	266.63
TJ 09	34	11.55	313.71
TJ 09	35	13.70	297.79
TJ 09	36	9.84	350.61
TJ 09	37	11.66	2.26
TJ 09	38	10.25	302.90
TJ 09	39	9.11	336.14
TJ 09	40	8.69	161.51
TJ 09	41	10.88	64.51
TJ 09	42	9.52	229.48
TJ 09	43	8.39	124.87
TJ 09	44	7.91	282.63
TJ 09	45	11.87	319.88
TJ 09	46	11.42	48.49
TJ 09	47	8.81	76.93
TJ 09	48	8.41	116.38
TJ 09	49	10.61	300.46
TJ 09	50	11.46	222.47
TJ 10	1	11.16	115.18
TJ 10	2	11.55	318.26
TJ 10	3	10.09	48.95
TJ 10	4	9.90	60.90
TJ 10	5	9.90	251.91
TJ 10	6	11.87	302.92
TJ 10	7	7.88	313.31
TJ 10	8	9.34	225.58
TJ 10	9	8.40	83.70
TJ 10	10	10.63	140.73
TJ 10	11	9.03	100.35
TJ 10	12	11.34	66.18

Differential exhumation of cratonic and non-cratonic lithosphere revealed by apatite fission track thermochronology along the Paramirim aulacogen, Brazil --- Authors: Ana Fonseca, Simone Cruz, Tiago Novo, Zhiyuan He, Johan De Grave

Sample	Track_ID	Length	C-axis angle
TJ 10	13	9.78	332.86
TJ 10	14	10.73	297.08
TJ 10	15	8.49	325.59
TJ 10	16	11.24	321.22
TJ 10	17	11.27	255.38
TJ 10	18	9.82	82.15
TJ 10	19	10.93	352.10
TJ 10	20	6.44	57.03
TJ 10	21	8.61	294.77
TJ 10	22	10.63	300.34
TJ 10	23	9.25	325.99
TJ 10	24	8.78	257.80
TJ 12	1	11.55	32.85
TJ 12	2	11.74	313.00
TJ 12	3	11.31	89.44
TJ 12	4	8.98	289.29
TJ 12	5	8.63	8.63
TJ 12	6	9.33	204.44
TJ 12	7	8.23	27.27
TJ 12	8	13.95	311.64
TJ 12	9	12.40	119.67
TJ 12	10	11.16	69.85
TJ 12	11	14.14	68.63
TJ 12	12	9.11	217.88
TJ 12	13	9.33	154.92
TJ 12	14	11.27	253.44
TJ 12	15	12.70	60.04
TJ 12	16	13.68	298.94
TJ 12	17	12.82	229.35
TJ 12	18	15.13	212.05
TJ 12	19	13.98	258.07
TJ 12	20	12.84	288.92
TJ 12	21	10.88	25.98
TJ 12	22	12.03	332.23
TJ 12	23	13.09	70.50
TJ 12	24	12.20	111.39
TJ 12	25	10.81	21.09
TJ 12	26	11.76	71.87
TJ 12	27	8.18	121.07
TJ 12	28	8.66	331.37
TJ 12	29	12.38	82.38
TJ 12	30	11.14	252.82
TJ 12	31	12.29	249.00
TJ 12	32	11.61	293.89
TJ 12	33	8.18	327.05
TJ 12	34	8.89	242.60
TJ 12	35	12.02	33.87
TJ 12	36	9.97	306.39
TJ 12	37	13.14	304.99
TJ 12	38	11.49	298.32
TJ 12	39	9.52	324.14
TJ 12	40	14.53	289.87
TJ 12	41	11.62	256.25
TJ 12	42	9.43	281.13
TJ 12	43	10.99	319.99
TJ 12	44	13.64	280.36
TJ 12	45	11.56	148.65
TJ 12	46	11.34	244.28
TJ 12	47	7.01	304.94
TJ 12	48	7.91	87.85
TJ 12	49	11.15	19.37
TJ 12	50	14.70	129.74
TJ 12	51	11.84	329.36
TJ 12	52	10.77	15.36
TJ 12	53	9.78	315.09
TJ 12	54	8.79	283.41
TJ 12	55	7.35	316.41
TJ 12	56	8.91	60.90
TJ 12	57	11.30	49.93
TJ 12	58	8.47	214.67
TJ 12	59	10.39	257.20
TJ 12	60	6.70	251.23
TJ 12	61	10.07	326.51
TJ 12	62	10.13	230.75
TJ 12	63	13.92	273.57
TJ 12	64	11.79	227.99
TJ 12	65	11.21	53.19
TJ 12	66	8.50	239.52
TJ 12	67	10.18	304.50
TJ 12	68	13.11	238.18
TJ 12	69	11.35	326.34
TJ 12	70	5.68	200.28
TJ 12	71	9.94	296.31
TJ 12	72	9.67	341.87
TJ 12	73	9.80	77.13
TJ 12	74	7.68	335.89
TJ 12	75	12.42	311.74
TJ 12	76	12.39	301.37
TJ 12	77	11.16	330.18
TJ 12	78	9.66	9.59

Sample	Track_ID	Length	C-axis angle
TJ 12	79	8.71	333.65
TJ 12	80	10.19	219.97
TJ 12	81	14.09	307.04
TJ 12	82	9.24	28.23
TJ 12	83	12.29	110.93
TJ 12	84	10.32	323.67
TJ 12	85	9.26	287.77
TJ 12	86	9.12	254.45
TJ 12	87	9.68	79.67
TJ 12	88	13.42	60.23
TJ 12	89	14.86	310.09
TJ 12	90	6.60	281.76
TJ 12	91	12.10	348.08
TJ 12	92	11.63	116.28
TJ 12	93	10.82	55.22
TJ 12	94	9.92	110.96
TJ 12	95	7.21	51.05
TJ 12	96	10.07	75.04
TJ 12	97	13.10	31.16
TJ 12	98	11.04	321.64
TJ 12	99	14.21	222.91
TJ 12	100	13.03	309.59
TJ 13	1	11.60	310.05
TJ 13	2	14.22	232.79
TJ 13	3	12.28	256.67
TJ 13	4	14.05	306.06
TJ 13	5	13.83	290.13
TJ 13	6	8.01	284.62
TJ 13	7	10.59	288.91
TJ 13	8	10.42	59.75
TJ 13	9	16.00	27.57
TJ 13	10	10.37	244.15
TJ 13	11	9.45	293.88
TJ 13	12	9.96	263.39
TJ 13	13	9.29	263.86
TJ 13	14	13.32	251.24
TJ 13	15	11.68	249.25
TJ 13	16	6.90	59.43
TJ 13	17	11.46	292.43
TJ 13	18	7.96	228.46
TJ 13	19	13.96	257.75
TJ 13	20	9.97	57.29
TJ 13	21	13.06	128.49
TJ 13	22	10.15	69.10
TJ 13	23	12.04	47.30
TJ 13	24	9.63	68.66
TJ 13	25	9.52	45.71
TJ 13	26	11.18	60.77
TJ 13	27	11.61	103.92
TJ 13	28	9.52	144.77
TJ 13	29	5.90	326.68
TJ 13	30	10.43	185.10
TJ 13	31	7.59	114.84
TJ 13	32	7.98	44.55
TJ 13	33	9.86	324.70
TJ 13	34	12.43	99.27
TJ 13	35	12.54	31.31
TJ 13	36	11.00	42.74
TJ 13	37	6.82	113.53
TJ 13	38	13.37	78.72
TJ 13	39	10.32	38.23
TJ 13	40	10.04	37.13
TJ 13	41	13.95	113.99
TJ 13	42	7.08	112.07
TJ 13	43	6.27	307.37
TJ 13	44	11.08	299.88
TJ 13	45	9.49	26.17
TJ 13	46	8.36	37.47
TJ 13	47	12.30	53.22
TJ 13	48	10.82	39.57
TJ 13	49	12.18	298.86
TJ 13	50	12.27	263.99
TJ 13	51	11.64	218.05
TJ 13	52	10.06	231.60
TJ 13	53	11.77	220.96
TJ 13	54	6.11	252.43
TJ 13	55	8.19	73.63
TJ 13	56	11.10	247.99
TJ 13	57	11.63	255.27
TJ 13	58	13.24	282.42
TJ 13	59	10.46	261.78
TJ 13	60	11.04	301.14
TJ 13	61	15.21	205.19
TJ 13	62	12.23	219.88
TJ 13	63	8.12	59.43
TJ 13	64	12.23	329.35
TJ 13	65	14.29	53.15
TJ 13	66	13.49	334.19
TJ 13	67	9.04	46.93
TJ 13	68	10.54	289.38

Sample	Track_ID	Length	C-axis angle
TJ 13	69	9.79	56.02
TJ 13	70	11.56	280.06
TJ 13	71	8.79	295.22
TJ 13	72	10.79	290.10
TJ 13	73	13.64	55.10
TJ 13	74	8.09	113.04
TJ 13	75	11.90	252.54
TJ 13	76	9.23	77.88
TJ 13	77	8.61	377.00
TJ 13	78	9.03	134.83
TJ 13	79	13.91	264.26
TJ 13	80	8.22	97.92
TJ 14	1	11.18	104.90
TJ 14	2	11.27	294.64
TJ 14	3	12.04	127.92
TJ 14	4	6.89	70.23
TJ 14	5	8.27	339.10
TJ 14	6	14.33	34.86
TJ 14	7	12.52	314.53
TJ 14	8	12.14	309.35
TJ 14	9	9.96	298.23
TJ 14	10	13.37	108.55
TJ 14	11	13.05	304.44
TJ 14	12	11.33	265.65
TJ 14	13	12.58	337.47
TJ 14	14	10.64	331.79
TJ 14	15	10.34	64.16
TJ 14	16	10.62	304.04
TJ 14	17	11.61	294.48
TJ 14	18	11.52	310.87
TJ 14	19	10.58	68.76
TJ 14	20	11.18	130.25
TJ 14	21	10.78	99.23
TJ 14	22	12.50	63.99
TJ 14	23	12.13	282.66
TJ 14	24	9.74	30.22
TJ 14	25	10.77	303.44
TJ 14	26	13.43	247.80
TJ 14	27	12.19	263.60
TJ 14	28	12.09	247.19
TJ 14	29	13.98	19.79
TJ 14	30	8.98	38.72
TJ 14	31	12.76	129.83
TJ 14	32	9.69	126.91
TJ 14	33	12.08	312.44

Sample	Track_ID	Length	C-axis angle
TJ 14	34	11.73	62.56
TJ 14	35	11.87	173.18
TJ 14	36	12.17	239.39
TJ 14	37	10.92	207.91
TJ 14	38	12.64	298.32
TJ 14	39	12.79	81.04
TJ 14	40	10.48	77.71
TJ 14	41	12.44	303.41
TJ 14	42	12.43	59.43
TJ 14	43	11.31	110.93
TJ 14	44	13.55	356.04
TJ 14	45	12.91	57.26
TJ 14	46	9.70	311.64
TJ 14	47	11.28	328.77
TJ 14	48	13.82	295.62
TJ 14	49	11.11	241.66
TJ 14	50	9.16	241.88
TJ 14	51	12.56	87.60
TJ 14	52	11.90	231.75
TJ 14	53	13.44	78.62
TJ 14	54	11.74	56.30
TJ 14	55	10.48	66.69
TJ 14	56	9.72	102.25
TJ 14	57	10.32	286.19
TJ 14	58	12.64	92.57
TJ 14	59	10.12	286.37
TJ 14	60	11.41	52.34
TJ 14	61	10.61	318.61
TJ 14	62	9.59	282.56
TJ 14	63	10.83	221.39
TJ 14	64	13.03	313.89
TJ 14	65	12.55	253.16
TJ 15	1	10.62	135.22
TJ 15	2	8.18	316.74
TJ 15	3	6.15	359.39
TJ 15	4	8.86	241.41
TJ 15	5	11.14	216.42
TJ 15	6	7.95	290.66
TJ 15	7	8.64	318.03
TJ 15	8	7.72	219.89
TJ 15	9	11.23	286.04
TJ 15	10	7.42	120.56
TJ 15	11	6.25	61.20
TJ 15	12	11.77	48.13
TJ 15	13	13.80	257.03

Sample	Track_ID	Length	C-axis angle
TJ 15	14	13.80	287.93
TJ 15	15	8.58	21.41
TJ 15	16	8.10	276.30
TJ 15	17	8.85	127.82
TJ 15	18	7.72	19.98
TJ 15	19	12.74	202.60
TJ 15	20	10.79	317.70
TJ 15	21	12.55	325.56
TJ 15	22	11.50	314.75
TJ 15	23	7.97	67.77
TJ 15	24	10.24	51.00
TJ 15	25	10.09	73.92
TJ 15	26	6.37	298.58
TJ 15	27	9.55	312.10
TJ 15	28	12.21	129.41
TJ 15	29	6.84	33.36
TJ 15	30	8.36	286.69
TJ 15	31	9.35	56.01
TJ 15	32	10.95	35.55
TJ 15	33	5.62	84.04
TJ 15	34	7.59	255.70
TJ 15	35	9.91	302.02
TJ 15	36	12.84	318.38
TJ 15	37	12.51	317.50
TJ 15	38	9.12	267.42
TJ 15	39	6.45	261.65
TJ 15	40	8.28	274.88
TJ 15	41	8.14	318.56
TJ 15	42	7.97	112.70
TJ 15	43	11.06	319.83
TJ 15	44	11.28	147.45
TJ 15	45	8.22	71.25
TJ 15	46	9.44	319.88
TJ 15	47	12.94	296.97
TJ 15	48	6.91	301.13
TJ 15	49	11.41	327.85
TJ 15	50	8.52	281.17
TJ 15	51	7.25	43.16
TJ 15	52	11.48	225.35
TJ 15	53	10.56	65.93
TJ 15	54	12.76	286.49
TJ 15	55	7.42	47.76
TJ 15	56	13.41	312.07
TJ 15	57	14.68	76.16
TJ 15	58	9.48	344.94

Sample	Track_ID	Length	C-axis angle
TJ 15	59	8.18	280.85
TJ 15	60	7.93	223.98
TJ 15	61	14.10	238.85
TJ 15	62	9.74	239.98
TJ 15	63	11.01	110.05
TJ 15	64	11.75	280.38
TJ 15	65	11.81	247.20
TJ 15	66	9.50	278.79
TJ 15	67	6.35	271.36
TJ 15	68	8.58	222.80
TJ 15	69	8.33	223.34
TJ 15	70	12.55	201.54
TJ 15	71	11.13	251.54
TJ 15	72	10.42	280.79
TJ 15	73	9.01	246.88
TJ 15	74	5.89	227.97
TJ 15	75	7.42	291.39
TJ 15	76	10.07	313.97
TJ 15	77	6.66	336.02
TJ 15	78	5.20	44.86
TJ 15	79	9.52	299.88
TJ 15	80	9.45	52.50
TJ 15	81	6.01	332.47
TJ 15	82	13.40	48.42
TJ 15	83	13.18	73.95
TJ 15	84	7.68	292.07
TJ 15	85	6.00	243.64
TJ 15	86	8.35	315.71
TJ 15	87	7.00	324.29
TJ 15	88	11.40	294.32
TJ 15	89	8.25	43.21
TJ 15	90	13.77	254.87
TJ 15	91	14.73	244.97
TJ 15	92	10.79	284.71
TJ 15	93	6.69	307.30
TJ 15	94	10.09	244.08
TJ 15	95	9.76	243.59
TJ 15	96	11.84	307.53
TJ 15	97	11.60	259.04
TJ 15	98	10.37	238.07
TJ 16	1	13.18	247.34
TJ 16	2	11.17	228.10
TJ 16	3	9.90	212.01
TJ 16	4	10.02	235.97
TJ 16	5	11.16	233.42

Differential exhumation of cratonic and non-cratonic lithosphere revealed by apatite fission track thermochronology along the Paramirim aulacogen, Brazil --- Authors: Ana Fonseca, Simone Cruz, Tiago Novo, Zhiyuan He, Johan De Grave

Sample	Track_ID	Length	C-axis angle	Sample	Track_ID	Length	C-axis angle
TJ 16	7	12.88	253.05	TJ 16	52	11.12	121.09
TJ 16	8	12.65	228.55	TJ 16	53	10.62	225.34
TJ 16	9	11.85	307.66	TJ 16	54	12.88	216.58
TJ 16	10	11.17	216.79	TJ 16	55	11.60	290.82
TJ 16	11	13.67	243.85	TJ 16	56	8.18	232.04
TJ 16	12	10.69	254.22	TJ 16	57	13.89	235.35
TJ 16	13	11.33	264.02	TJ 16	58	13.88	237.53
TJ 16	14	11.13	237.59	TJ 16	59	11.65	240.03
TJ 16	15	9.06	302.62	TJ 16	60	11.91	300.18
TJ 16	16	10.48	286.75	TJ 16	61	9.22	75.25
TJ 16	17	11.42	237.01	TJ 16	62	12.21	309.79
TJ 16	18	12.80	214.85	TJ 16	63	13.98	314.95
TJ 16	19	10.53	307.18	TJ 16	64	12.01	59.90
TJ 16	20	11.62	225.22	TJ 16	65	9.58	104.59
TJ 16	21	10.87	253.59	TJ 16	66	11.51	300.21
TJ 16	22	10.97	248.31	TJ 16	67	9.59	49.31
TJ 16	23	10.04	215.54	TJ 16	68	9.50	61.24
TJ 16	24	10.96	214.48	TJ 16	69	12.92	139.56
TJ 16	25	13.25	277.57	TJ 16	70	9.08	348.84
TJ 16	26	14.45	304.38	TJ 16	71	13.47	18.50
TJ 16	27	13.66	289.69	TJ 16	72	11.60	259.22
TJ 16	28	13.97	64.74	TJ 16	73	8.68	298.69
TJ 16	29	14.49	50.05	TJ 16	74	10.90	215.33
TJ 16	30	13.10	62.38	TJ 16	75	13.17	288.65
TJ 16	31	10.81	92.24	TJ 16	76	14.70	311.82
TJ 16	32	13.20	46.72	TJ 16	77	16.22	35.04
TJ 16	33	12.62	69.11	TJ 16	78	14.79	68.76
TJ 16	34	10.16	84.84	TJ 16	79	14.23	110.82
TJ 16	35	13.40	78.97	TJ 16	80	13.75	232.90
TJ 16	36	12.50	119.85	TJ 16	81	14.11	60.51
TJ 16	37	12.01	48.79	TJ 16	82	9.97	272.82
TJ 16	38	8.98	120.08	TJ 16	83	13.65	51.17
TJ 16	39	13.91	205.69	TJ 16	84	12.94	281.06
TJ 16	40	8.83	278.45	TJ 16	85	10.38	107.02
TJ 16	41	13.07	278.25	TJ 16	86	12.40	46.48
TJ 16	42	11.89	248.35	TJ 16	87	12.98	102.71
TJ 16	43	13.75	222.37	TJ 16	88	11.56	55.32
TJ 16	44	13.93	279.51	TJ 16	89	11.10	45.60
TJ 16	45	13.60	109.03	TJ 16	90	10.98	273.89
TJ 16	46	10.33	199.96	TJ 16	91	11.25	244.46
TJ 16	47	10.08	284.49	TJ 16	92	9.44	242.06
TJ 16	48	10.61	225.40	TJ 16	93	14.65	213.73
TJ 16	49	12.08	276.91	TJ 16	94	11.15	288.68
TJ 16	50	10.04	257.13	TJ 16	95	11.66	232.83

Sample	Track_ID	Length	C-axis angle
TJ 16	97	10.54	293.00
TJ 16	98	15.86	253.88
TJ 16	99	12.78	296.74
TJ 16	100	14.52	233.34
TJ 17	1	11.04	256.01
TJ 17	2	12.27	259.76
TJ 17	3	11.17	247.20
TJ 17	4	13.54	295.25
TJ 17	5	12.35	235.80
TJ 17	6	9.67	228.18
TJ 17	7	8.94	333.75
TJ 17	8	12.58	33.12
TJ 17	9	12.25	302.87
TJ 17	10	12.23	52.00
TJ 17	11	12.14	297.17
TJ 17	12	10.82	222.50
TJ 17	13	12.22	314.08
TJ 17	14	11.83	258.12
TJ 17	15	8.79	240.06
TJ 17	16	7.73	212.59
TJ 17	17	9.74	303.59
TJ 17	18	12.92	303.44
TJ 17	19	10.36	247.64
TJ 17	20	9.91	325.06
TJ 17	21	11.95	225.59
TJ 17	22	13.69	246.06
TJ 17	23	12.72	313.84
TJ 17	24	13.84	213.28
TJ 17	25	8.20	338.98
TJ 17	26	8.82	330.35
TJ 17	27	10.31	309.11
TJ 17	28	11.02	218.55
TJ 17	29	9.17	247.87
TJ 17	30	12.05	306.79
TJ 17	31	11.03	247.78
TJ 17	32	10.30	232.86
TJ 17	33	11.26	220.05
TJ 17	34	8.89	299.60
TJ 17	35	11.82	316.98
TJ 17	36	12.20	299.56
TJ 17	37	11.71	321.09
TJ 17	38	11.81	331.83
TJ 17	39	9.00	358.62
TJ 17	40	11.94	34.67

Sample	Track_ID	Length	C-axis angle
TJ 17	42	9.14	323.76
TJ 17	43	8.91	258.60
TJ 17	44	10.04	31.70
TJ 17	45	10.98	126.54
TJ 17	46	11.59	296.44
TJ 17	47	11.67	138.51
TJ 17	48	12.34	120.11
TJ 17	49	10.86	236.55
TJ 17	50	10.59	285.07
TJ 17	51	10.43	298.05
TJ 17	52	13.63	306.98
TJ 17	53	12.27	250.45
TJ 17	54	10.19	238.19
TJ 17	55	9.60	310.59
TJ 17	56	10.44	222.03
TJ 17	57	9.06	306.27
TJ 17	58	11.40	59.70
TJ 17	59	11.08	281.40
TJ 17	60	10.28	299.54
TJ 17	61	7.78	156.83
TJ 17	62	12.58	249.35
TJ 17	63	10.23	232.66
TJ 17	64	10.11	123.86
TJ 17	65	11.36	114.27
TJ 17	66	11.70	45.11
TJ 17	67	11.19	252.15
TJ 17	68	11.16	288.26
TJ 17	69	11.83	59.92
TJ 17	70	12.97	297.01
TJ 17	71	11.97	55.17
TJ 17	72	9.35	108.56
TJ 17	73	9.11	300.00
TJ 17	74	12.08	56.46
TJ 17	75	10.72	307.89
TJ 17	76	11.56	35.53
TJ 17	77	9.58	94.25
TJ 17	78	10.26	30.18
TJ 17	79	12.81	43.77
TJ 17	80	14.27	68.32
TJ 17	81	11.69	41.47
TJ 17	82	12.71	285.46
TJ 17	83	9.75	271.20
TJ 17	84	9.99	42.88
TJ 17	85	11.60	12.71

Sample	Track_ID	Length	C-axis angle
TJ 17	87	8.85	55.27
TJ 17	88	10.90	32.73
TJ 17	89	13.67	98.08
TJ 17	90	9.47	47.51
TJ 17	91	11.97	110.53
TJ 17	92	9.27	9.43
TJ 17	93	13.08	27.82
TJ 17	94	12.41	4.03
TJ 17	95	11.87	321.39
TJ 17	96	11.87	48.39
TJ 17	97	12.22	70.21
TJ 17	98	13.22	119.79
TJ 17	99	8.34	282.43
TJ 17	100	8.37	50.33
TJ 18	1	8.61	48.11
TJ 18	2	12.78	327.34
TJ 18	3	13.86	72.39
TJ 18	4	9.34	112.86
TJ 18	5	12.50	123.34
TJ 18	6	14.59	41.56
TJ 18	7	12.54	330.87
TJ 18	8	10.01	232.43
TJ 18	9	10.24	287.84
TJ 18	10	10.23	300.44
TJ 18	11	9.73	299.04
TJ 18	12	12.08	172.11
TJ 18	13	13.79	224.42
TJ 18	14	11.30	320.76
TJ 18	15	13.10	341.39
TJ 18	16	14.22	332.84
TJ 18	17	9.87	81.20
TJ 18	18	12.82	94.82
TJ 18	19	8.37	21.53
TJ 18	20	12.59	43.12
TJ 18	21	11.87	292.19
TJ 18	22	12.97	134.21
TJ 18	23	13.00	64.81
TJ 18	24	11.81	167.48
TJ 18	25	12.16	86.92
TJ 18	26	11.53	21.85
TJ 18	27	11.25	320.24
TJ 18	28	14.37	129.56
TJ 18	29	12.30	343.02
TJ 18	30	12.02	100.46

Sample	Track_ID	Length	C-axis angle
TJ 18	32	10.64	334.52
TJ 18	33	12.51	107.01
TJ 18	34	11.58	111.96
TJ 18	35	9.42	50.25
TJ 18	36	9.52	41.45
TJ 18	37	11.32	110.07
TJ 18	38	12.82	310.71
TJ 18	39	11.01	304.14
TJ 18	40	9.02	54.19
TJ 18	41	13.78	39.57
TJ 18	42	10.75	225.15
TJ 18	43	13.14	291.61
TJ 18	44	12.97	295.69
TJ 18	45	7.76	238.32
TJ 18	46	14.57	72.42
TJ 18	47	13.81	303.20
TJ 18	48	11.03	68.97
TJ 18	49	10.89	77.89
TJ 18	50	10.50	289.74
TJ 18	51	10.22	9.09
TJ 18	52	11.00	258.02
TJ 18	53	9.48	260.63
TJ 18	54	12.43	240.06
TJ 18	55	13.39	61.71
TJ 18	56	9.11	305.74
TJ 18	57	11.39	30.35
TJ 18	58	10.90	290.28
TJ 18	59	12.71	56.19
TJ 18	60	10.38	112.29
TJ 18	61	9.85	28.52
TJ 18	62	11.38	344.25
TJ 18	63	13.42	296.11
TJ 18	64	12.27	256.78
TJ 18	65	11.63	321.18
TJ 18	66	14.10	295.74
TJ 18	67	13.61	265.66
TJ 18	68	10.14	75.00
TJ 18	69	12.48	322.98
TJ 18	70	11.68	294.42
TJ 18	71	9.67	213.26
TJ 18	72	13.18	289.63
TJ 18	73	10.26	67.70
TJ 18	74	12.62	303.86
TJ 18	75	13.86	39.42

Differential exhumation of cratonic and non-cratonic lithosphere revealed by apatite fission track thermochronology along the Paramirim aulacogen, Brazil --- Authors: Ana Fonseca, Simone Cruz, Tiago Novo, Zhiyuan He, Johan De Grave

Sample	Track_ID	Length	C-axis angle
TJ 18	77	9.79	313.97
TJ 18	78	13.91	288.30
TJ 18	79	14.85	315.24
TJ 18	80	11.19	302.31
TJ 18	81	11.77	62.66
TJ 18	82	11.17	302.29
TJ 18	83	9.12	302.13
TJ 18	84	11.36	299.19
TJ 18	85	14.13	315.56
TJ 18	86	11.80	255.10
TJ 18	87	9.78	257.55
TJ 18	88	10.55	252.99
TJ 18	89	12.97	324.96
TJ 18	90	12.53	315.33
TJ 18	91	11.63	248.91
TJ 18	92	11.60	247.03
TJ 18	93	12.00	192.80
TJ 18	94	10.55	240.62
TJ 18	95	14.73	311.70
TJ 18	96	12.81	299.70
TJ 18	97	10.61	215.15
TJ 18	98	10.78	193.77
TJ 18	99	9.71	301.38
TJ 18	100	11.71	60.16
TJ 20	1	13.75	69.76
TJ 20	2	11.50	198.92
TJ 20	3	10.34	349.52
TJ 20	4	12.06	307.52
TJ 20	5	10.01	300.91
TJ 20	6	10.62	183.82
TJ 20	7	10.72	173.59
TJ 20	8	10.18	348.13
TJ 20	9	11.41	282.86
TJ 20	10	11.25	228.79
TJ 20	11	9.85	272.07
TJ 20	12	9.21	292.30
TJ 20	13	13.56	299.37
TJ 20	14	13.59	299.22
TJ 20	15	12.31	14.35
TJ 20	16	9.66	240.93
TJ 20	17	7.44	293.89
TJ 20	18	13.08	230.16
TJ 20	19	9.03	200.34
TJ 20	20	8.95	318.62

Sample	Track_ID	Length	C-axis angle
TJ 20	22	10.89	299.85
TJ 20	23	10.02	348.84
TJ 20	24	10.42	281.15
TJ 20	25	10.82	290.34
TJ 20	26	10.05	114.23
TJ 20	27	12.76	61.01
TJ 20	28	9.32	28.44
TJ 20	29	9.90	77.29
TJ 20	30	8.85	62.43
TJ 20	31	12.62	117.26
TJ 20	32	10.50	212.86
TJ 20	33	9.49	95.49
TJ 20	34	13.01	338.76
TJ 20	35	13.08	45.71
TJ 20	36	8.69	71.05
TJ 20	37	13.16	308.15
TJ 20	38	9.93	210.69
TJ 20	39	9.23	215.78
TJ 20	40	10.53	217.96
TJ 20	41	9.81	312.60
TJ 20	42	11.17	316.55
TJ 20	43	11.80	244.26
TJ 20	44	10.07	221.98
TJ 20	45	8.97	249.96
TJ 20	46	10.24	248.44
TJ 20	47	7.95	239.17
TJ 20	48	12.02	251.42
TJ 20	49	11.11	304.97
TJ 20	50	11.98	220.61
TJ 20	51	11.42	317.46
TJ 20	52	9.04	23.96
TJ 20	53	11.71	50.35
TJ 20	54	12.62	315.13
TJ 20	55	12.88	280.10
TJ 20	56	11.48	28.00
TJ 20	57	11.73	57.10
TJ 20	58	11.39	224.99
TJ 20	59	11.40	300.76
TJ 20	60	11.41	213.24
TJ 20	61	10.32	208.57
TJ 20	62	9.62	290.57
TJ 20	63	11.19	244.55
TJ 20	64	14.91	255.92
TJ 20	65	12.24	304.61

Differential exhumation of cratonic and non-cratonic lithosphere revealed by apatite fission track thermochronology along the Paramirim aulacogen, Brazil --- Authors: Ana Fonseca, Simone Cruz, Tiago Novo, Zhiyuan He, Johan De Grave

Sample	Track_ID	Length	C-axis angle
TJ 20	67	10.13	233.10
TJ 20	68	13.34	27.37
TJ 20	69	12.30	221.38
TJ 20	70	11.53	323.52
TJ 20	71	13.54	232.03
TJ 20	72	9.80	294.50
TJ 20	73	12.63	17.88
TJ 20	74	14.62	335.95
TJ 20	75	10.40	313.05
TJ 20	76	9.53	294.36
TJ 20	77	12.41	302.68
TJ 20	78	10.79	237.68
TJ 20	79	9.86	224.26
TJ 20	80	12.00	323.24
TJ 20	81	14.85	298.09
TJ 20	82	12.26	231.34
TJ 20	83	11.38	231.28
TJ 20	84	14.21	297.76
TJ 20	85	12.92	299.02
TJ 20	86	10.61	28.22
TJ 20	87	11.96	293.00
TJ 20	88	13.14	229.87
TJ 20	89	13.19	237.84
TJ 20	90	13.60	305.77
TJ 20	91	8.67	307.13
TJ 20	92	13.55	221.35
TJ 20	93	11.98	256.89
TJ 20	94	12.80	329.30
TJ 20	95	9.98	258.09
TJ 20	96	10.73	219.03
TJ 20	97	12.11	315.75
TJ 20	98	10.58	295.34
TJ 20	99	13.22	285.44
TJ 20	100	11.31	20.26
TJ 21	1	13.21	302.93
TJ 21	2	12.84	55.13
TJ 21	3	14.68	209.62
TJ 21	4	13.86	238.41
TJ 21	5	14.36	281.70
TJ 21	6	7.72	297.80
TJ 21	7	12.97	251.47
TJ 21	8	9.73	284.56
TJ 21	9	9.82	213.06
TJ 21	10	13.28	317.22

Sample	Track_ID	Length	C-axis angle
TJ 21	12	13.48	263.91
TJ 21	13	13.54	8.84
TJ 21	14	13.14	16.01
TJ 21	15	13.22	109.64
TJ 21	16	13.03	101.83
TJ 21	17	13.44	134.32
TJ 21	18	12.20	300.92
TJ 21	19	9.84	44.70
TJ 21	20	9.85	109.61
TJ 21	21	11.76	61.18
TJ 21	22	12.77	325.23
TJ 21	23	10.47	320.03
TJ 21	24	9.89	333.46
TJ 21	25	11.90	137.46
TJ 21	26	11.00	255.80
TJ 21	27	14.99	323.30
TJ 21	28	10.16	213.91
TJ 21	29	11.35	243.14
TJ 21	30	12.98	232.01
TJ 21	31	13.04	288.63
TJ 21	32	13.52	247.78
TJ 21	33	10.71	326.33
TJ 21	34	13.19	317.51
TJ 21	35	11.53	140.29
TJ 21	36	12.41	227.61
TJ 21	37	12.60	299.11
TJ 21	38	12.02	107.85
TJ 21	39	10.23	216.58
TJ 21	40	14.27	15.07
TJ 21	41	11.50	297.36
TJ 21	42	10.10	236.01
TJ 21	43	11.26	234.56
TJ 21	44	8.59	313.67
TJ 21	45	12.85	296.99
TJ 21	46	10.93	240.81
TJ 21	47	13.72	278.33
TJ 21	48	8.00	226.73
TJ 21	49	12.22	271.29
TJ 21	50	13.07	254.79
TJ 21	51	15.03	253.18
TJ 21	52	11.79	260.68
TJ 21	53	12.60	308.23
TJ 21	54	12.03	330.99
TJ 21	55	11.35	310.41

Differential exhumation of cratonic and non-cratonic lithosphere revealed by apatite fission track thermochronology along the Paramirim aulacogen, Brazil --- Authors: Ana Fonseca, Simone Cruz, Tiago Novo, Zhiyuan He, Johan De Grave

Sample	Track_ID	Length	C-axis angle	Sample	Track_ID	Length	C-axis angle
TJ 21	57	10.39	53.47	TJ 22	2	10.13	295.38
TJ 21	58	8.27	328.67	TJ 22	3	10.91	323.02
TJ 21	59	14.81	74.95	TJ 22	4	11.04	291.66
TJ 21	60	10.45	32.58	TJ 22	5	12.92	43.07
TJ 21	61	11.47	106.98	TJ 22	6	11.98	291.37
TJ 21	62	8.65	293.41	TJ 22	7	10.99	73.58
TJ 21	63	14.98	307.96	TJ 22	8	14.07	43.39
TJ 21	64	12.68	241.95	TJ 22	9	13.05	293.24
TJ 21	65	12.65	300.98	TJ 22	10	12.11	297.53
TJ 21	66	11.06	300.20	TJ 22	11	11.00	321.10
TJ 21	67	9.27	293.31	TJ 22	12	14.86	332.39
TJ 21	68	11.98	254.64	TJ 22	13	15.07	352.62
TJ 21	69	9.04	257.95	TJ 22	14	13.18	336.14
TJ 21	70	13.29	285.39	TJ 22	15	10.75	70.75
TJ 21	71	11.65	279.47	TJ 22	16	9.82	7.03
TJ 21	72	14.10	25.06	TJ 22	17	10.71	305.59
TJ 21	73	13.51	326.63	TJ 22	18	12.68	233.50
TJ 21	74	13.27	46.25	TJ 22	19	10.39	234.30
TJ 21	75	11.09	25.65	TJ 22	20	13.41	286.60
TJ 21	76	12.18	251.37	TJ 22	21	10.50	302.78
TJ 21	77	10.88	294.20	TJ 22	22	8.66	59.23
TJ 21	78	12.89	239.36	TJ 22	23	10.79	292.74
TJ 21	79	13.91	291.40	TJ 22	24	10.33	298.64
TJ 21	80	13.67	58.77	TJ 22	25	14.75	356.95
TJ 21	81	13.63	226.39	TJ 22	26	12.61	60.36
TJ 21	82	10.92	285.28	TJ 22	27	12.41	337.30
TJ 21	83	12.29	319.04	TJ 22	28	9.20	311.35
TJ 21	84	10.31	41.78	TJ 22	29	12.57	299.95
TJ 21	85	13.28	260.15	TJ 22	30	11.73	326.56
TJ 21	86	12.07	304.69	TJ 22	31	10.14	256.35
TJ 21	87	13.18	239.08	TJ 22	32	11.83	244.52
TJ 21	88	11.52	250.59	TJ 22	33	12.47	345.55
TJ 21	89	12.82	273.96	TJ 22	34	9.54	262.85
TJ 21	90	13.20	229.56	TJ 22	35	11.40	305.84
TJ 21	91	11.15	207.96	TJ 22	36	13.89	315.84
TJ 21	92	13.69	317.63	TJ 22	37	10.22	286.61
TJ 21	93	11.42	286.76	TJ 22	38	12.54	249.87
TJ 21	94	9.96	279.52	TJ 22	39	12.14	249.39
TJ 21	95	11.77	241.78	TJ 22	40	12.25	335.11
TJ 21	96	11.40	253.99	TJ 22	41	13.88	244.39
TJ 21	97	13.95	294.21	TJ 22	42	13.32	252.95
TJ 21	98	10.67	253.64	TJ 22	43	14.20	262.48
TJ 21	99	12.60	294.37	TJ 22	44	12.47	295.44
TJ 21	100	14.41	291.81	TJ 22	45	10.96	35.96

Sample	Track_ID	Length	C-axis angle
TJ 22	47	12.04	292.26
TJ 22	48	13.85	17.07
TJ 22	49	10.63	320.34
TJ 22	50	15.33	339.25
TJ 22	51	13.61	287.70
TJ 22	52	14.65	255.39
TJ 22	53	13.51	324.71
TJ 22	54	13.77	65.85
TJ 22	55	13.21	298.11
TJ 22	56	10.71	233.90
TJ 23	1	14.36	78.08
TJ 23	2	11.06	331.23
TJ 23	3	12.01	309.34
TJ 23	4	13.29	298.82
TJ 23	5	13.57	321.57
TJ 23	6	13.32	306.18
TJ 23	7	14.46	13.21
TJ 23	8	14.77	320.50
TJ 23	9	14.17	55.04
TJ 23	10	12.56	329.84
TJ 23	11	12.66	339.77
TJ 23	12	12.89	77.58
TJ 23	13	11.87	320.43
TJ 23	14	12.99	284.38
TJ 23	15	12.51	307.69
TJ 23	16	13.52	292.51
TJ 23	17	12.06	311.09
TJ 23	18	13.23	53.51
TJ 23	19	14.48	65.53
TJ 23	20	12.95	285.64
TJ 23	21	12.58	253.11
TJ 23	22	12.34	324.28
TJ 23	23	12.97	40.65
TJ 23	24	12.36	76.01
TJ 23	25	13.92	326.80
TJ 23	26	11.79	55.46
TJ 23	27	12.03	297.52
TJ 23	28	13.44	327.58
TJ 23	29	12.87	37.34
TJ 23	30	12.84	291.08
TJ 23	31	13.79	307.12
TJ 23	32	13.96	70.41
TJ 23	33	12.14	280.11
TJ 23	34	12.90	51.34

Sample	Track_ID	Length	C-axis angle
TJ 23	36	12.34	236.32
TJ 23	37	10.49	259.79
TJ 23	38	12.85	316.63
TJ 23	39	12.48	286.46
TJ 23	40	12.62	316.13
TJ 23	41	12.31	299.58
TJ 23	42	11.39	255.55
TJ 23	43	12.45	277.27
TJ 23	44	13.16	213.26
TJ 23	45	11.44	316.67
TJ 23	46	11.72	63.93
TJ 23	47	13.11	49.60
TJ 23	48	12.78	108.97
TJ 23	49	12.92	305.37
TJ 23	50	14.58	309.77
TJ 23	51	13.63	70.43
TJ 23	52	15.77	43.67
TJ 23	53	12.71	70.86
TJ 23	54	12.22	53.92
TJ 23	55	11.55	349.45
TJ 23	56	10.48	43.99
TJ 23	57	13.41	63.67
TJ 23	58	10.68	61.32
TJ 23	59	11.30	47.37
TJ 23	60	10.91	54.18
TJ 23	61	12.30	104.68
TJ 23	62	14.21	296.87
TJ 23	63	12.15	344.59
TJ 23	64	14.75	297.19
TJ 23	65	10.07	55.88
TJ 23	66	12.16	264.71
TJ 23	67	13.05	287.68
TJ 23	68	14.29	197.88
TJ 23	69	13.33	289.82
TJ 23	70	12.79	257.41
TJ 23	71	11.10	277.54
TJ 23	72	13.96	257.66
TJ 23	73	11.21	230.78
TJ 23	74	13.43	234.84
TJ 23	75	13.26	277.34
TJ 23	76	14.15	258.29
TJ 23	77	11.61	298.07
TJ 23	78	13.02	242.75
TJ 23	79	13.88	278.93

Sample	Track_ID	Length	C-axis angle
TJ 23	81	11.63	45.18
TJ 23	82	12.42	21.26
TJ 23	83	13.35	126.31
TJ 23	84	14.29	141.03
TJ 23	85	11.58	137.70
TJ 23	86	11.55	132.43
TJ 23	87	15.11	316.56
TJ 23	88	12.72	49.26
TJ 23	89	14.60	62.50
TJ 23	90	13.09	118.80
TJ 23	91	14.51	108.40
TJ 23	92	12.68	314.92
TJ 23	93	13.24	70.41
TJ 23	94	9.06	316.47
TJ 23	95	13.52	306.17
TJ 23	96	10.34	316.57
TJ 23	97	10.43	314.13
TJ 23	98	13.22	322.62
TJ 23	99	11.08	235.13
TJ 23	100	14.24	305.51
TJ 24	1	11.23	320.62
TJ 24	2	14.70	324.50
TJ 24	3	12.64	36.95
TJ 24	4	11.98	67.63
TJ 24	5	14.61	329.72
TJ 24	6	12.35	114.07
TJ 24	7	13.21	320.33
TJ 24	8	9.69	248.51
TJ 24	9	11.01	248.24
TJ 24	10	9.74	255.37
TJ 24	11	14.60	228.10
TJ 24	12	14.39	65.00
TJ 24	13	11.47	37.66
TJ 24	14	11.66	239.20
TJ 24	15	11.01	190.26
TJ 24	16	9.78	296.69
TJ 24	17	12.53	300.16
TJ 24	18	12.22	225.63
TJ 24	19	12.02	192.20
TJ 24	20	11.54	312.50
TJ 24	21	11.35	280.36
TJ 24	22	9.84	249.78
TJ 24	23	11.88	249.37
TJ 24	24	9.86	225.31

Sample	Track_ID	Length	C-axis angle
TJ 24	26	12.52	310.19
TJ 24	27	11.28	71.12
TJ 24	28	10.62	64.29
TJ 24	29	10.45	311.29
TJ 24	30	12.23	294.79
TJ 24	31	14.20	352.63
TJ 24	32	11.46	331.73
TJ 24	33	12.66	232.25
TJ 24	34	9.77	295.49
TJ 24	35	12.91	308.35
TJ 24	36	12.60	294.81
TJ 24	37	9.04	238.22
TJ 24	38	11.12	293.11
TJ 24	39	12.89	305.25
TJ 24	40	14.23	225.42
TJ 24	41	10.94	291.73
TJ 24	42	12.61	110.09
TJ 24	43	10.53	301.84
TJ 24	44	11.82	75.26
TJ 24	45	10.17	255.05
TJ 24	46	11.95	326.76
TJ 24	47	9.83	309.75
TJ 24	48	14.85	326.61
TJ 24	49	9.64	102.22
TJ 24	50	9.32	140.15
TJ 24	51	14.69	65.94
TJ 24	52	11.95	81.94
TJ 24	53	9.38	108.36
TJ 25	1	11.32	221.11
TJ 25	2	11.53	220.71
TJ 25	3	10.50	305.54
TJ 25	4	10.67	286.26
TJ 25	5	10.76	74.28
TJ 25	6	14.26	316.20
TJ 25	7	14.36	293.58
TJ 25	8	13.14	228.66
TJ 25	9	10.55	259.17
TJ 25	10	13.22	214.71
TJ 25	11	12.22	239.01
TJ 25	12	11.21	259.04
TJ 25	13	12.21	238.87
TJ 25	14	11.98	226.81
TJ 25	15	13.80	285.12
TJ 25	16	11.76	308.85

Sample	Track_ID	Length	C-axis angle
TJ 25	18	11.82	314.10
TJ 25	19	9.55	209.26
TJ 25	20	11.66	254.81
TJ 25	21	14.53	289.74
TJ 25	22	10.22	294.94
TJ 25	23	10.89	249.46
TJ 25	24	10.69	280.25
TJ 25	25	12.96	226.85
TJ 25	26	9.60	307.57
TJ 25	27	13.06	341.66
TJ 25	28	13.04	253.77
TJ 25	29	13.65	284.87
TJ 25	30	10.84	44.57
TJ 25	31	13.34	61.35
TJ 25	32	13.45	304.10
TJ 25	33	13.39	336.85
TJ 25	34	13.03	319.48
TJ 25	35	13.82	229.59
TJ 25	36	9.88	317.74
TJ 25	37	9.75	35.66
TJ 25	38	15.52	300.82
TJ 25	39	12.34	317.73
TJ 25	40	14.39	299.93
TJ 25	41	10.88	287.69
TJ 25	42	11.65	305.40
TJ 25	43	11.66	230.38
TJ 25	44	13.11	247.36
TJ 25	45	14.77	301.87
TJ 25	46	11.03	218.27
TJ 25	47	13.17	287.04
TJ 25	48	12.38	237.93
TJ 25	49	12.69	259.42
TJ 25	50	12.64	216.79
TJ 25	51	13.50	288.63
TJ 25	52	13.93	262.29
TJ 25	53	12.09	298.02
TJ 25	54	10.05	293.43
TJ 25	55	10.28	282.00
TJ 25	56	9.65	41.52
TJ 25	57	10.56	327.03
TJ 25	58	13.17	290.63
TJ 25	59	10.29	297.64
TJ 25	60	9.87	67.72
TJ 25	61	12.70	29.83
TJ 25	63	11.68	240.41
TJ 25	64	11.23	239.83
TJ 25	65	12.42	298.87
TJ 25	66	12.43	326.21
TJ 25	67	11.56	260.91
TJ 25	68	11.57	39.20
TJ 25	69	13.91	59.73
TJ 25	70	11.45	63.57
TJ 25	71	14.29	61.22
TJ 25	72	12.02	233.09
TJ 25	73	12.54	227.88
TJ 25	74	14.21	284.45
TJ 25	75	12.17	237.17
TJ 25	76	13.44	290.00
TJ 25	77	10.86	300.73
TJ 25	78	14.10	318.43
TJ 25	79	11.03	300.55
TJ 25	80	10.59	79.55
TJ 25	81	13.27	25.57
TJ 25	82	10.85	300.77
TJ 25	83	12.81	67.96
TJ 25	84	12.57	345.25
TJ 25	85	11.18	71.96
TJ 25	86	14.22	322.37
TJ 25	87	12.02	243.71
TJ 25	88	11.30	292.17
TJ 25	89	12.43	253.29
TJ 25	90	11.25	293.49
TJ 25	91	15.48	249.82
TJ 25	92	12.06	312.09
TJ 25	93	12.81	291.92
TJ 25	94	11.96	313.22
TJ 25	95	13.25	291.68
TJ 25	96	14.13	302.48
TJ 25	97	12.74	284.90
TJ 25	98	14.02	314.50
TJ 25	99	12.17	55.37
TJ 25	100	13.78	42.10
TJ 26	1	9.07	328.17
TJ 26	2	8.65	253.83
TJ 26	3	14.48	270.15
TJ 26	4	13.15	214.99
TJ 26	5	10.54	105.79
TJ 26	6	12.70	285.99

Differential exhumation of cratonic and non-cratonic lithosphere revealed by apatite fission track thermochronology along the Paramirim aulacogen, Brazil --- Authors: Ana Fonseca, Simone Cruz, Tiago Novo, Zhiyuan He, Johan De Grave

Sample	Track_ID	Length	C-axis angle
TJ 26	8	10.80	225.10
TJ 26	9	8.44	248.50
TJ 26	10	10.62	284.32
TJ 26	11	10.24	296.33
TJ 26	12	12.39	241.62
TJ 26	13	12.01	308.61
TJ 26	14	11.59	260.67
TJ 26	15	14.04	246.11
TJ 26	16	12.87	247.96
TJ 26	17	12.24	253.03
TJ 26	18	11.12	167.54
TJ 26	19	10.80	181.70
TJ 26	20	11.19	290.08
TJ 26	21	11.84	276.50
TJ 26	22	12.52	41.79
TJ 26	23	9.44	285.20
TJ 26	24	13.85	124.95
TJ 26	25	10.47	65.80
TJ 26	26	10.40	47.20
TJ 26	27	9.45	285.29
TJ 26	28	9.81	262.42
TJ 26	29	13.84	222.87
TJ 26	30	10.90	287.00
TJ 26	31	10.65	316.55
TJ 26	32	12.18	298.54
TJ 26	33	11.58	72.87
TJ 26	34	10.95	313.89
TJ 26	35	10.61	67.53
TJ 26	36	11.14	320.35
TJ 26	37	12.93	307.59
TJ 26	38	9.65	320.43
TJ 26	39	10.16	285.86
TJ 26	40	13.09	50.87
TJ 26	41	13.52	64.88
TJ 26	42	13.14	20.18
TJ 26	43	9.99	100.91
TJ 26	44	11.07	254.14
TJ 26	45	14.68	117.73
TJ 26	46	10.91	292.50
TJ 26	47	12.45	51.77
TJ 26	48	11.03	252.81
TJ 26	49	11.56	39.10
TJ 26	50	12.66	229.47
TJ 26	51	11.92	245.11
TJ 26	53	12.82	230.83
TJ 26	54	10.07	232.43
TJ 26	55	9.28	58.39
TJ 26	56	10.46	132.15
TJ 26	57	13.79	45.31
TJ 26	58	11.18	316.12
TJ 26	59	12.67	71.61
TJ 26	60	10.96	50.65
TJ 26	61	11.94	292.40
TJ 26	62	13.29	332.60
TJ 26	63	10.54	60.38
TJ 26	64	11.79	50.61
TJ 26	65	10.57	318.04
TJ 26	66	10.92	281.71
TJ 26	67	11.94	50.95
TJ 26	68	12.08	77.16
TJ 26	69	12.41	326.17
TJ 26	70	10.56	20.56
TJ 26	71	12.01	107.37
TJ 26	72	12.35	48.71
TJ 26	73	10.65	73.80
TJ 26	74	11.33	278.60
TJ 26	75	10.31	77.96
TJ 26	76	14.61	72.12
TJ 26	77	9.69	54.16
TJ 26	78	12.47	112.42
TJ 26	79	12.78	26.89
TJ 26	80	14.03	73.54
TJ 26	81	12.69	68.04
TJ 26	82	13.01	41.78
TJ 26	83	11.49	30.83
TJ 26	84	11.74	55.96
TJ 26	85	10.80	239.78
TJ 26	86	11.05	260.00
TJ 26	87	11.35	244.30
TJ 26	88	8.14	254.57
TJ 26	89	11.52	293.65
TJ 26	90	9.33	253.51
TJ 26	91	12.56	327.15
TJ 26	92	13.52	241.44
TJ 26	93	7.87	317.49
TJ 26	94	10.84	255.34
TJ 26	95	13.28	285.74
TJ 26	96	10.72	319.99

Differential exhumation of cratonic and non-cratonic lithosphere revealed by apatite fission track thermochronology along the Paramirim aulacogen, Brazil --- Authors: Ana Fonseca, Simone Cruz, Tiago Novo, Zhiyuan He, Johan De Grave

Sample	Track_ID	Length	C-axis angle	Sample	Track_ID	Length	C-axis angle
TJ 26	98	11.34	296.25	TJ 29	15	13.83	121.96
TJ 26	99	11.42	210.80	TJ 29	16	10.98	122.77
TJ 26	100	12.47	136.06	TJ 29	17	12.98	306.70
TJ 28	1	12.43	55.85	TJ 29	18	13.00	118.39
TJ 28	2	10.36	60.55	TJ 29	19	12.89	313.78
TJ 28	3	11.64	76.64	TJ 29	20	13.13	301.78
TJ 28	4	10.32	305.65	TJ 29	21	12.83	114.39
TJ 28	5	12.30	289.05	TJ 29	22	10.66	51.30
TJ 28	6	9.21	300.18	TJ 29	23	14.40	308.01
TJ 28	7	8.60	75.16	TJ 29	24	9.60	62.14
TJ 28	8	11.00	73.92	TJ 29	25	8.20	32.38
TJ 28	9	10.52	291.95	TJ 29	26	12.06	287.32
TJ 28	10	8.98	280.42	TJ 29	27	11.39	287.23
TJ 28	11	12.37	297.96	TJ 29	28	10.77	314.02
TJ 28	12	10.89	244.34	TJ 29	29	10.24	343.63
TJ 28	13	9.49	59.82	TJ 29	30	12.47	306.88
TJ 28	14	10.89	68.01	TJ 29	31	13.23	48.99
TJ 28	15	12.14	314.00	TJ 29	32	12.16	37.41
TJ 28	16	11.65	244.89	TJ 29	33	8.68	233.36
TJ 28	17	12.90	279.39	TJ 29	34	10.24	10.24
TJ 28	18	11.84	247.90	TJ 29	35	11.02	63.12
TJ 28	19	10.43	30.55	TJ 29	36	11.00	35.69
TJ 28	20	11.13	239.09	TJ 29	37	13.58	253.92
TJ 28	21	11.39	303.92	TJ 29	38	13.28	258.11
TJ 28	22	13.11	232.28	TJ 29	39	11.92	308.45
TJ 28	23	9.15	296.77	TJ 29	40	13.64	38.90
TJ 28	24	9.97	229.64	TJ 29	41	14.13	296.73
TJ 28	25	10.61	253.04	TJ 29	42	10.18	312.50
TJ 28	26	12.31	308.88	TJ 29	43	11.57	271.77
TJ 28	27	11.21	253.94	TJ 29	44	11.44	247.62
TJ 28	28	10.91	314.29	TJ 29	45	12.92	294.55
TJ 29	1	11.96	347.00	TJ 29	46	11.36	45.34
TJ 29	2	10.39	76.62	TJ 29	47	13.59	288.81
TJ 29	3	12.16	340.79	TJ 29	48	11.72	109.67
TJ 29	4	13.47	327.97	TJ 29	49	9.03	64.78
TJ 29	5	14.37	42.26	TJ 29	50	12.12	75.72
TJ 29	6	9.34	316.55	TJ 29	51	13.83	52.22
TJ 29	7	12.96	323.78	TJ 29	52	13.75	304.07
TJ 29	8	7.71	228.74	TJ 29	53	12.77	63.90
TJ 29	9	12.57	224.13	TJ 29	54	11.34	320.67
TJ 29	10	9.09	278.24	TJ 29	55	11.53	278.78
TJ 29	11	11.61	253.97	TJ 29	56	13.71	284.68
TJ 29	12	14.44	303.09	TJ 29	57	13.39	71.39
TJ 29	13	10.59	289.97	TJ 29	58	13.11	336.16

Differential exhumation of cratonic and non-cratonic lithosphere revealed by apatite fission track thermochronology along the Paramirim aulacogen, Brazil --- Authors: Ana Fonseca, Simone Cruz, Tiago Novo, Zhiyuan He, Johan De Grave

Sample	Track_ID	Length	C-axis angle
TJ 29	60	7.96	76.18
TJ 29	61	13.56	145.40
TJ 29	62	13.59	81.81
TJ 29	63	12.78	68.04
TJ 29	64	12.71	66.38
TJ 29	65	12.93	290.14
TJ 29	66	13.00	58.34
TJ 29	67	13.94	29.61
TJ 29	68	10.67	313.63
TJ 29	69	13.08	36.49
TJ 29	70	12.60	322.58
TJ 29	71	10.60	284.17
TJ 29	72	12.17	158.36
TJ 29	73	11.73	297.47
TJ 29	74	12.68	235.00
TJ 29	75	13.92	295.58
TJ 29	76	13.56	249.84
TJ 29	77	14.48	152.01
TJ 29	78	13.11	289.22
TJ 29	79	11.58	225.36
TJ 29	80	11.40	322.37
TJ 29	81	12.95	64.16
TJ 29	82	11.34	53.95
TJ 29	83	13.70	59.14
TJ 29	84	12.21	309.76
TJ 29	85	12.58	78.33
TJ 29	86	11.17	297.33
TJ 29	87	11.98	27.39
TJ 29	88	8.64	296.13
TJ 29	89	12.91	287.68
TJ 29	90	10.31	69.63
TJ 29	91	14.41	282.99
TJ 29	92	10.95	39.19
TJ 29	93	12.32	116.70
TJ 29	94	7.48	310.87
TJ 29	95	9.37	24.80
TJ 29	96	11.83	115.98
TJ 29	97	12.96	71.58
TJ 29	98	8.90	126.58
TJ 29	99	12.69	66.56
TJ 29	100	13.32	256.27
TJ 31	1	11.69	295.78
TJ 31	2	11.08	53.23
TJ 31	3	9.35	319.03
TJ 31	5	13.39	67.26
TJ 31	6	11.83	62.26
TJ 31	7	10.84	64.64
TJ 31	8	11.26	55.34
TJ 31	9	11.33	83.42
TJ 31	10	12.56	305.87
TJ 31	11	11.92	118.60
TJ 31	12	10.16	43.71
TJ 31	13	12.72	318.78
TJ 31	14	10.64	6.18
TJ 31	15	9.90	319.24
TJ 31	16	11.53	315.39
TJ 31	17	12.13	234.97
TJ 31	18	10.81	215.86
TJ 31	19	10.47	251.93
TJ 31	20	11.51	310.80
TJ 31	21	11.07	303.53
TJ 31	22	12.65	47.64
TJ 31	23	9.07	294.30
TJ 31	24	9.38	255.27
TJ 31	25	9.01	235.77
TJ 31	26	10.89	290.42
TJ 31	27	10.76	21.28
TJ 31	28	9.13	317.48
TJ 31	29	11.93	302.01
TJ 31	30	8.82	242.42
TJ 31	31	11.33	250.75
TJ 31	32	12.70	290.19
TJ 31	33	11.48	225.48
TJ 31	34	13.12	255.07
TJ 31	35	11.46	249.18
TJ 31	36	10.85	259.60
TJ 31	37	11.87	296.11
TJ 31	38	9.10	283.22
TJ 31	39	11.00	56.68
TJ 31	40	10.87	40.90
TJ 31	41	13.26	338.79
TJ 31	42	10.58	255.37
TJ 31	43	10.03	267.63
TJ 31	44	12.08	314.00
TJ 31	45	12.95	236.25
TJ 31	46	12.62	207.71
TJ 31	47	11.88	215.11
TJ 31	48	10.55	216.08

Sample	Track_ID	Length	C-axis angle
TJ 31	50	10.76	318.77
TJ 31	51	9.75	239.48
TJ 31	52	10.41	297.32
TJ 31	53	11.06	243.93
TJ 31	54	10.30	288.96
TJ 31	55	10.29	250.02
TJ 31	56	11.30	270.23
TJ 31	57	12.29	245.06
TJ 31	58	11.06	267.93
TJ 31	59	11.01	297.44
TJ 31	60	10.86	285.52
TJ 31	61	10.15	244.78
TJ 31	62	9.98	302.61
TJ 31	63	13.18	193.04
TJ 31	64	8.82	326.41
TJ 31	65	8.98	227.30
TJ 31	66	9.85	318.80
TJ 31	67	10.80	255.10
TJ 31	68	11.33	247.96
TJ 31	69	9.26	316.20
TJ 31	70	9.36	302.88
TJ 31	71	9.59	251.72
TJ 31	72	9.15	308.97
TJ 31	73	10.25	43.52
TJ 31	74	10.81	225.00
TJ 31	75	9.50	292.18
TJ 31	76	11.00	351.60

Sample	Track_ID	Length	C-axis angle
TJ 31	78	10.53	234.19
TJ 31	79	9.69	252.72
TJ 31	80	11.39	258.33
TJ 31	81	11.87	67.00
TJ 31	82	11.45	30.01
TJ 31	83	8.97	264.95
TJ 31	84	9.03	283.53
TJ 31	85	9.94	289.58
TJ 31	86	10.55	54.45
TJ 31	87	12.48	342.18
TJ 31	88	9.73	292.12
TJ 31	89	7.45	297.94
TJ 31	90	10.20	240.55
TJ 31	91	12.19	252.66
TJ 31	92	9.16	149.09
TJ 31	93	11.54	235.31
TJ 31	94	9.92	227.99
TJ 31	95	8.32	26.72
TJ 31	96	10.81	33.14
TJ 31	97	9.59	275.48
TJ 31	98	11.87	323.34
TJ 31	99	10.43	293.72
TJ 31	100	11.75	296.82

1. Gallagher, K. Transdimensional inverse thermal history modeling for quantitative thermochronology. *J. Geophys. Res.* **117**, 1–16 (2012).

2. Green, P. F., Duddy, I. R., Gleadow, A. J. W., Tingate, P. R. & Laslett, G. M. Thermal annealing of fission tracks in apatite. *Chem. Geol. Isot. Geosci. Sect.* **59**, 237–253 (1986).
